# Alcohol Exposure *In Utero* and Child Academic Achievement^*^

**DOI:** 10.1111/ecoj.12144

**Published:** 2014-05-23

**Authors:** Stephanie von Hinke Kessler Scholder, George L Wehby, Sarah Lewis, Luisa Zuccolo

**Affiliations:** University of York; University of Iowa and NBER; University of Bristol; University of Bristol

## Abstract

We examine the effect of prenatal alcohol exposure on child academic achievement. We use a genetic variant in the maternal alcohol-metabolism gene *ADH1B* to instrument for alcohol exposure, whilst controlling for the child's genotype on the same variant. We show that the instrument is unrelated to an extensive range of parental characteristics and behaviour. OLS regressions suggest an ambiguous association between alcohol exposure and attainment but there is a strong social gradient in drinking, with mothers in higher socio-economic groups more likely to drink. In contrast to the OLS, the IV estimates show clear negative effects of prenatal alcohol exposure.

The first scientific study that examined the effects of excessive alcohol intake during pregnancy was published by a Liverpool prison physician in 1899 (Sullivan, 1899). He argued that alcohol consumption caused the higher rates of stillbirth observed among female alcoholic prisoners compared to their sober counterparts. The detrimental effects of excessive drinking during pregnancy are currently well known. The effects of low-to-moderate drinking, however, are less conclusive. Indeed, there are conflicting recommendations regarding the ‘threshold’ for maternal prenatal alcohol consumption, ranging from total abstinence in most countries including the US to restricted consumption in the UK. Only in 1995 did the UK Department of Health issue guidelines for women who were (planning to become) pregnant, stating that ‘women should not drink more than 1 or 2 units of alcohol once or twice a week, and should avoid episodes of intoxication’ (Department of Health, 1995). Their most recent guidelines are very similar: despite advising pregnant women not to drink in the first three months of pregnancy, they mention that, if women choose to drink, they should not exceed 1–2 units once or twice a week, as ‘at this low level, there is no evidence of any harm to the unborn baby’ (NICE, 2008).

These conflicting recommendations arise from inconsistent findings in observational studies of the correlation between low-to-moderate alcohol consumption and child development (including physical and mental health, cognitive and behavioural outcomes). Some find negative effects on child development, some do not find evidence of developmental differences and others argue that it improves child outcomes (for reviews of this literature, see Abel and Hannigan (1995); Polygenis *et al*. (1998); Gray and Henderson (2006). One of the major problems in estimating the causal effects of prenatal alcohol consumption is that it is a choice; as such, it may be related to other unobserved characteristics that also affect the outcome of interest, biasing the estimates.

This article examines the impact of alcohol exposure *in utero*, as proxied by whether the mother consumed any alcohol during pregnancy, on child academic achievement. We also investigate the effect of the dose, pattern and duration of exposure. We deal with unobserved residual confounding using ‘Mendelian randomisation’, referring to the random allocation of an individual's genotype at conception (Davey Smith and Ebrahim, 2003). Although this allocation is random at the family trio level (i.e. from parents to children), at a population level it has been shown that genetic variants are largely unrelated to the many socio-economic and behavioural characteristics that are closely linked with each other and that confound conventional observational studies. This has been shown using a wide range of genetic variants,^1^ different data sources,^2^ and for an extensive set of background characteristics;^3^ see Bhatti *et al*. (2005), WTCCC (2007), Davey Smith *et al*. (2008), Kivimäki *et al*. (2008), von Hinke Kessler Scholder *et al*. (2011) and Lawlor *et al*. (2013).^4^ Hence, we employ a carefully validated genetic variant that is associated with decreased alcohol exposure as an instrumental variable (IV) for exposure to alcohol *in utero* (Zuccolo *et al*., 2009). Under assumptions discussed in detail below, genetic variants are independent of unobservable confounders, including those that occur *in utero*. As such, Mendelian randomisation can be exploited to make causal inferences about the effects of behavioural or health conditions that have (at least partly) a genetic aetiology on certain outcomes of interest. For a brief introduction to some of the genetic terms referred to in this study, see Appendix A.

Our contribution to the literature is, first, to examine the causal effects of prenatal alcohol exposure on child development. As suggested by the relatively few studies attempting to investigate the causal effects (see below), it is particularly difficult to estimate these due to unobserved residual confounding. Second, as it is obviously unethical to design a randomised controlled trial, we show that quasi-experimental designs, such as Mendelian randomisation, may provide a powerful and useful alternative for causal inference. We also present a thorough discussion of the assumptions required in Mendelian randomisation experiments, and provide additional evidence on the validity of genetic variants as instrumental variables by testing its correlation with an unusually wide range of maternal and paternal characteristics and behaviour. Third, we add to the literature on the long-term effects of the early environment on later child outcomes; for a recent overview, see Almond and Currie (2011), on potential differential investments by parents in response to child development (Almond and Mazumder, 2013), as well as on identifying important periods of parental investments *per se* (Cunha and Heckman, 2007). Finally, we provide advice to policy makers, distinguishing between the effects of low-to-moderate *versus* excessive alcohol exposure *in utero*.

We start by presenting some simple descriptive statistics about the prevalence of maternal prenatal alcohol consumption, as these are not well documented in the economics literature. We show that 63% of pregnant women drink at some point during pregnancy, with 17% reporting that they binged (defined as drinking four units of alcohol in a day). On average, women drink 1.5 units of alcohol per week. OLS regressions show an ambiguous association between alcohol exposure *in utero* and children's educational attainment, with exposure to wine having a positive association, but exposure to beer being negative. Binge drinking is bad for the child but a longer exposure to alcohol (in terms of the number of trimesters) is positively associated with the child's outcomes.

We then present evidence of a strong social gradient in alcohol exposure, with older mothers and those of higher socio-economic position being more likely to drink during pregnancy and, particularly, drink wine. Beer consumption, on the other hand, is associated with smoking, lower education and worse mental health. We use a genetic variant in the maternal Alcohol Dehydrogenase 1B gene, an alcohol-metabolising gene, as an instrument for prenatal alcohol exposure. We show that the single nucleotide polymorphism (SNP) is associated with alcohol exposure *in utero*. In addition, we demonstrate that it is not related to any of the background characteristics that we show to be associated with prenatal drinking. To provide additional evidence on the validity of our IV approach, we exploit the richness of our data and correlate the SNP to an unusually extensive range of maternal and paternal prenatal characteristics and behaviours; we find no evidence of any systematic associations that would suggest the instrument is invalid. In stark contrast to the OLS, our IV estimates show strong negative effects of alcohol exposure *in utero* on child educational achievement, which are robust to a large set of model specifications. In addition, the reduced form regressions show that the effects are solely driven by the maternal SNP, with no impact of the child's SNP on the child's academic attainment. The results also suggest that low-to-moderate (as opposed to excessive) exposure may have similar negative effects on child outcomes. Yet, despite the large negative effects, we find little evidence of differential parental responsive investments to child development, exploring an unusually wide range of parental post-natal responses and behaviour.

The relatively few studies in the economics literature that have attempted to deal with unobserved confounding related to prenatal alcohol exposure generally find large negative effects on child development.^5^ Exploiting a Swedish alcohol policy experiment from the 1960s that increased alcohol availability in two Swedish counties by allowing grocery stores to sell strong beer, Nilsson (2008) investigates the effects of prenatal alcohol exposure on a set of adult outcomes. The policy experiment led to a sharp increase in alcohol consumption in the experimental regions, particularly among youths, causing the experiment to be discontinued prematurely. Using a difference-in-difference-in-differences design, he finds that children born to mothers under age 21, who have the longest prenatal exposure to the experiment at delivery, have a lower human capital attainment later in life: total years of schooling are reduced by 0.27 on average, with males being more affected (0.47 years) than females (0.10 years). Children exposed prenatally to alcohol are 4 percentage points less likely to have completed high school, and 2.5 percentage points less likely to have graduated. Their earnings at age 32 are 24% lower compared to those not exposed, and the proportion on welfare increased by 5 percentage points.

Wüst (2010) uses Danish survey and register data to examine the effect of maternal inputs on child birth outcomes (birth weight, foetal growth and preterm birth). OLS analyses suggest an ambiguous association between prenatal alcohol consumption and birth outcomes. The sibling fixed effects, however, show clear negative effects, suggesting that each daily unit of alcohol decreases birth weight by 147 g (4%) and increases the probability of a preterm birth by 7.8 percentage points.^6^ Exploiting changes in the minimum legal drinking age over time across US states, similar adverse effects on birth outcomes are reported by Fertig and Watson (2009), whilst Barreca and Page (2012) find no effects. Finally, Zhang (2010) examines the relationship between state-level alcohol taxes, prenatal drinking and infant health using the US Natality Files and the behavioural risk surveillance system. The results suggest that an increase in taxes on beer relates to a decrease in the incidence of low birth weight.

Our article is structured as follows: the next Section reviews the mechanisms through which alcohol can affect the foetus, and discusses the metabolism of alcohol. Section Methodological Framework presents the methodological framework and discusses the validity of the instrument. The data are introduced in Section Data, followed by the results in Section Results. We conclude with a discussion of our findings.

## Mechanisms

### In Utero Alcohol Exposure and Child Development

Excessive drinking during pregnancy is well known to be detrimental to the foetus, potentially leading to foetal alcohol spectrum disorder (FASD, a pattern of mental and physical defects). The effects of low-to-moderate drinking are less clear and there is no consensus as to what level of exposure is toxic to the foetus.

Numerous mechanisms have been suggested to contribute to alcohol-induced foetal damage. Its effects on the developing brain are particularly complex, as – depending on the developmental stage of the cells – alcohol can affect cell division, the survival of migrating cells, the establishment of mature cell structures/functions, as well as interfere with the brain's cellular functions. For example, after multiplication through cell division, nerve cells in the foetal brain migrate to an appropriate location where they mature to their full form and function. Alcohol exposure during cell division may decrease the cell division rate, whilst exposure during later stages may deplete cells due to alcohol-induced cell death (Goodlett and Horn, 2001). Hence, the timing of alcohol exposure may be important for different aspects of brain development. However, because the brain is one of the first organs to begin and the last to complete development, it is susceptible to damage throughout pregnancy (Guerri, 2002). Furthermore, as it is the blood alcohol level, rather than the amount of alcohol consumed, that can cause foetal damage, binge drinking is generally regarded as more damaging than drinking the same amount of alcohol over a longer period (Guerri, 2002).

Any damage due to prenatal alcohol exposure, however, does not necessarily show at birth or in infancy but may only manifest later in childhood, adolescence or even adulthood. Hence, affected children may go undetected until problems arise in the academic environment (Coles *et al*., 1991), with neurodevelopmental problems potentially persisting into adult life (Gray and Henderson, 2006). The most prominent dysfunctions include deficits in verbal learning and in integrating visual information, alterations in spatial memory and in reaction time, impaired attention, reduced academic achievement and other cognitive and motor skills (Russell, 1991; Guerri, 2002).

### The Metabolism of Alcohol

**Figure 1:**
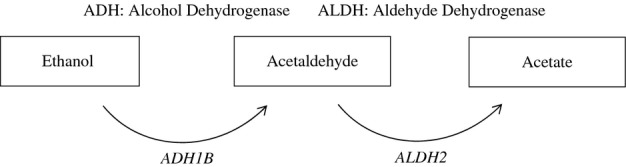
The Metabolism of Alcohol

Figure1 graphically presents the first two steps in the metabolism of ethanol.^7^ The alcohol dehydrogenase (ADH) family of enzymes, which includes ADH1B, catalyses its first step: oxidising ethanol to acetaldehyde, a mutagenic and carcinogenic metabolite. With that, the ADH1B enzyme plays a major role in the breakdown of ethanol. The rare variant of rs1229984, a single nucleotide polymorphism, or SNP, in the ADH1B gene, greatly increases ADH1B enzymatic activity, resulting in a quicker reduction of blood alcohol levels and sharper rises of acetaldehyde in blood and organs (see Appendix A for a brief introduction to some of the genetic terms used here). The latter in turn leads to symptoms such as increased heart rate and nausea. Individuals with the rare variant of *ADH1B* therefore consume less alcohol, as found in numerous studies across many populations (see below). Hence, foetuses of mothers who carry the rare variant of *ADH1B* have a reduced exposure to alcohol compared to foetuses of mothers who carry the common variant. Note that the effects of *ADH1B* on alcohol consumption are subtle: it does not make an individual an alcoholic or in other ways alcohol-dependent. Instead, it only reduces alcohol intake by a small amount.^8^

## Methodological Framework

We use a SNP in the alcohol dehydrogenase 1B (*ADH1B*) gene rs1229984 to explain variation in alcohol exposure *in utero*. The vast majority of individuals of European ancestry are homozygous for the common allele. In fact, there are very few individuals who are homozygous for the rare allele (<1%). We therefore specify a binary instrument, equalling 1 when the individual carries either one or two copies of the rare allele (A), assuming a dominant genetic model (as in Zuccolo *et al*. (2009); Zuccolo (2010)); that is, we compare individuals with genotype GA or AA to those with genotype GG.

### Potential Outcomes Framework

Let *Z*_*i*_ denote this binary genetic variant, with *Z*_*i*_ = 1 indicating that the mother of child *i* carries the rare variant, and *Z*_*i*_ = 0 implying that the mother of child *i* does not carry the rare variant. Let *A* and *S* denote random variables representing, respectively, alcohol intake and the educational outcome. Let *A*_*i*_(*z*) be the potential alcohol exposure for child *i* when the instrument is set to *z*. Similarly, let *S*_*i*_(*z*, *a*) be the potential outcome for child *i* that would be obtained if the instrument was set to *z* and alcohol exposure, the treatment variable, was set to *a*. Only one of the two potential exposures or treatments [*A*_*i*_(0) and *A*_*i*_(1)] and only one of the two potential outcomes 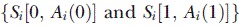 are ever observed for any one child.

As implicit in our notation, we assume that there is no interference between units (the Stable Unit Treatment Value Assumption, see Rubin, 1980). Given the set of potential outcomes, we can define the causal effect for child *i* of *Z* on *A* as 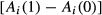 and the causal effect for child *i* of *Z* on *S* as 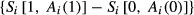. These are also known as the intention-to-treat effects. Our framework follows the work by, among others, Imbens and Angrist (1994) and Angrist *et al*. (1996). We briefly lay out our structural assumptions, and discuss more specifically how these apply to our research question.

Assumption (Conditional) Independence


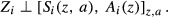


Independence assumes that the instrument is as good as randomly assigned. *Conditional* independence implies that independence holds conditional on some (vector of) covariate(s) *X*_*i*_, which would be denoted by *Z*_*i*_ ⊥ [*S*_*i*_(*z*, *a*), *A*_*i*_(*z*)]_*z*,*a*_ | *X*_*i*_.

Although genetic variants are randomly assigned at conception, the independence assumption can be violated when a systematic relationship exists between the allele frequency and the outcome of interest in different sub-populations; this is also known as population stratification. The most common example, and one that is important in the case of *ADH1B*, is ancestry. The *ADH1B* variant is one of the most ethnically stratified: the minor allele frequency (MAF; the frequency with which the rare allele occurs in the population) ranges from 2–5% in Western European populations to 60–70% in North-East Asia (Borinskaya *et al*., 2009). However, population stratification is likely to be less important in our study, as our data are collected in a small geographically defined region with a predominantly white population. In addition, we only include a child if the mother describes herself and the child's father as white and we adjust for 10 ancestry-informative principal components derived from analysis of the genomewide association data (Bouaziz *et al*., 2011). In subsection Descriptive Statistics, we evaluate the independence assumption by exploring the distribution of an extensive range of background characteristics by the value of the instrument. If the instrument is randomised, there should be no systematic differences in such characteristics.

Assumption Exclusion


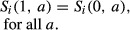


Exclusion implies that the instrument can only affect the outcome via its effect on *A*. Hence, we can write *S*_*i*_(*a*, *z*) = *S*_*i*_(*a*). If the exclusion restriction only holds conditional on *X*_*i*_, we may specify the exclusion restriction conditional on these covariate(s).

The exclusion restriction can in principle be violated in different situations. First, we need to consider the mechanism through which the variant affects alcohol exposure. This mechanism is relatively well understood, as discussed in subsection The Metabolism of Alcohol. Furthermore, we know that the *ADH1B* gene is predominantly expressed in the liver and (less so) in the lining of the stomach (Lee *et al*., 2006). The liver functions as the main organ in ethanol clearance; the stomach and small intestine are the principal absorption sites of ingested alcohol (Cortot *et al*., 1986).

Second, the exclusion restriction may be violated by pleiotropy, referring to the possibility that a SNP has multiple phenotypic associations. The gene expression and the well-understood mechanisms of *ADH1B* decrease the likelihood that *ADH1B* directly influences behaviours other than alcohol consumption. However, we cannot rule this out. It may be possible, for example, that carriers of the *ADH1B* rare allele are more likely to become anxious due to, or take medication to counter, any negative side effects of their alcohol intake, which in turn could directly affect foetal development, violating the exclusion restriction. We directly investigate this in subsection Descriptive Statistics, examining the distribution of an extensive set of maternal characteristics during pregnancy by genotype, including maternal diet, health and health conditions, physical activity, the use of medication, substance use, mental health and the use of chemicals.

Third, linkage disequilibrium (LD) refers to certain genetic variants potentially being co-inherited with other variants. Whether this violates the exclusion restriction depends on the function(s) of any co-inherited variants. *ADH1B* is in weak LD with other variants on the alcohol dehydrogenase genes, such as *ADH1A* and *ADH1C*, but these have all been shown to relate to alcohol metabolism, rather than to behaviours other than drinking (Birley *et al*., 2009).

More generally, we investigate the potential violation of the exclusion restriction by searching the medical literature on the relationships between *ADH1B* and other variables. In addition to consistent evidence of an association between *ADH1B* and alcohol intake (see also below), the SNP is consistently associated with conditions such as liver cirrhosis (Lorenzo *et al*., 2006), head and neck cancer (Brennan *et al*., 2004; McKay *et al*., 2011), upper aerodigestive tract cancer (Canova *et al*., 2009) and oesophageal cancer (Zhang *et al*., 2010). These are all associated with alcohol consumption, strongly suggesting that the SNP affects the outcomes through its effect on alcohol intake.

Assumption Non-zero effect of instrument on treatment


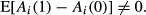


This implies that the instrument has some effect on treatment. It is essential for this association to be replicated in a large number of independent studies, as it has been shown that many initial genetic associations fail to replicate in independent samples (Colhoun *et al*., 2003; see also Beauchamp *et al*., 2011). Individuals with the rare variant of *ADH1B* are predicted to consume less alcohol than those with two common alleles. With that, foetuses of mothers who carry the rare variant have a reduced exposure to alcohol compared to foetuses of mothers who carry the common variant. This negative association is very robust and has been replicated in numerous independent genetic studies (Reich *et al*., 1998; Whitfield *et al*., 1998; Saccone *et al*., 2000, 2005; Loew *et al*., 2003; Wall *et al*., 2005; Duranceaux *et al*., 2006; Luo *et al*., 2006; Zintzaras *et al*., 2006; Zhang *et al*., 2007; Ghosh *et al*., 2008; Tolstrup *et al*., 2008; Macgregor *et al*., 2009; Sherva *et al*., 2009; Zuccolo *et al*., 2009), confirming Assumption 3; we show the standard statistical tests below.

Assumption Monotonicity


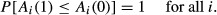


This means that the potential exposure or treatment for child *i* whose mother does not carry the rare variant is at least as high as the potential treatment for the same child whose mother does carry the rare variant, for all *i*. As discussed above, *ADH1B* does not make individuals alcoholics, nor does it stop people from drinking altogether; it only affects intake by a small amount. As such, individuals will not be aware of their genotype, and it is therefore very unlikely that they would engage in any potential ‘compensatory responses’, such as drinking less because they may be genetically less ‘protected’ against drinking. Hence, we assume that the foetus is less exposed to alcohol if the mother carries the risk allele than if she does not.

We use Assumptions 1–4 to interpret differences in average outcomes and treatments at different values of the instrument. Under these assumptions, the instrumental variables (Wald) estimand, defined as the ratio of the difference in average outcomes at two values of the instrument to the difference in average treatment at the same two values of the instrument, can be written as:

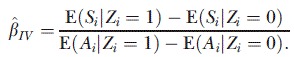
(1)

This is a local average treatment effect: the effect of *in utero* alcohol exposure on child academic achievement for children whose mother was induced by the instrument to reduce her alcohol intake. Our instrument picks up differences in children's exposure for mothers with and without the rare variant. Mothers who carry the rare variant are more likely to abstain in pregnancy, less likely to binge and on average consume less if they drink at all. We therefore start by exploring the effects of any alcohol exposure on academic achievement, but we are also interested in the effects of the dose, pattern and duration of exposure. However, estimating the effects of these additional treatments has implications for our IV approach. Indeed, with only one instrument, as we can only estimate the effect of one treatment at a time. When estimating the effect of an increase in the duration, for example, the exclusion restriction implies that our instrument *Z* only affects the outcome through its effect on the duration. However, *Z* may also affect the outcome through its effect on the dose and pattern of exposure. As such, specifying separate models for each treatment may violate our assumptions. In an attempt to deal with this, we start the analyses by defining treatment as a binary indicator equal to one if the foetus was exposed to any alcohol during the course of the pregnancy and equal to zero otherwise. This measure picks up a combined effect of any alcohol exposure *in utero*, ranging from light to heavy exposure, and including shorter as well as longer exposures.

We then estimate the effects of the dose, pattern and duration of exposure, but recognise the potential limitation of this approach with respect to the IV assumptions. The pattern variable (binge drinking) is binary; the dose and duration are count variables. Using a variable treatment intensity for the dose and duration, the Wald estimand becomes


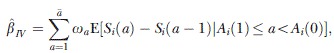


where 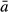 is the maximum of *a*, and the weights 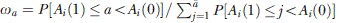 are non-negative and sum to one (Angrist and Imbens, 1995; Angrist and Pischke, 2009). Hence, the IV estimate with variable treatment intensity is a weighted average of the causal responses to a unit change in treatment, for those whose treatment status is affected by the instrument. The weight attached to the average of 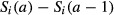 is proportional to the number of people who, because of the instrument, change their treatment intensity from more than *a* units to *a* or less (Angrist and Imbens, 1995). We show these weight functions in subsection Descriptive Statistics.

### Interpretation of the Estimates

The interpretation of our estimates is not straightforward, but rather depends on two important issues. First, we note that we identify an ‘overall’ or ‘total’ effect of alcohol exposure, which includes any effects that alcohol has on other substance use that in turn may affect child development. Indeed, if we were interested in the effects of alcohol exposure *per se*, our estimates may be either upward or downward biased, depending on whether alcohol and other substances are compliments or substitutes. For example, if alcohol and, for example, cannabis are substitutes (DiNardo and Lemieux, 2001) and prenatal exposure to cannabis negatively affects the child academic attainment *S*, the positive numerator of (1) will be reduced by the negative effect of cannabis. As the denominator is unchanged, the IV estimate would underestimate the effect of alcohol *per se*. Conversely, if alcohol and, for example, smoking are complements (Dee, 1999) and prenatal exposure to smoking negatively affects child development, the IV estimate would overestimate the effect of alcohol.

We directly explore any potential complements and substitutes of alcohol below, where we test whether there are any systematic differences by genotype in the use of a wide range of substances, including caffeine, smoking, cannabis, amphetamine, barbiturate, cocaine, heroin, methadone and ecstasy. We also examine whether maternal prenatal alcohol consumption affects her substance use using IV regressions. Our results show no systematic patterns, suggesting that the ‘overall’ effect we identify is similar to the ‘alcohol-effect’ *per se*.

The second issue to note regarding the interpretation of the estimates is that our treatment of interest is prenatal alcohol exposure. Foetal exposure to alcohol consists of three components: maternal consumption, maternal metabolism and foetal metabolism. The rare allele of maternal *ADH1B* rs1229984 is negatively associated with exposure through maternal consumption and metabolism: it is associated with a reduction in intake and an increase in the metabolic rate. Hence, the numerator of the Wald estimand (1) captures this total, or combined, ‘exposure effect’.

Ideally, therefore, we would like our treatment variable in the denominator of (1) to be a direct measure of exposure, such as foetal blood alcohol levels. For obvious practical and ethical reasons, however, we do not observe this. As we discuss below, we only observe one component of alcohol exposure: maternal alcohol consumption. This could be problematic, as, holding alcohol intake constant, blood alcohol levels may be lower in rare allele carriers of *ADH1B* due to the increased speed with which ethanol is broken down.

We search the literature to investigate the relative importance of the three components through which *ADH1B* may affect foetal alcohol exposure. As we discuss above, this shows clear evidence that *ADH1B* is an important determinant of the first component: alcohol intake. We also find this in our data: as we show below, those who carry the rare allele drink around 0.8 units a week less compared to those not carrying the rare allele; a difference similar to a 53% decrease relative to the mean. In addition, as alcohol consumed by the mother can cross the placenta without delay, it may immediately affect the foetus. Although there is no evidence on the importance of the effect of *ADH1B* on foetal metabolism, there is some – albeit little – evidence on adult metabolism. Neumark *et al*. (2004) find that *ADH1B* explains 8.5% of the variance in alcohol elimination rate in a sample of 109 ( Jewish) male students. Hence, although the evidence is limited, this would suggest that maternal metabolic rates do play a role, which we are not able to account for. In other words, as we only observe one of the three components of alcohol exposure in the denominator of (1), and as the numerator captures the full ‘exposure effect’, the IV estimate based on consumption alone is likely to be overestimated. Hence, although the sign of our estimates is correct, we cannot identify the exact magnitude, and we argue that our analysis provides an upper bound of the causal effect of alcohol exposure *in utero*.

## Data

Our data are from a cohort of children born in one geographic area (Avon) of England. Women eligible for enrolment in the population-based Avon Longitudinal Study of Parents and Children (ALSPAC) had an expected delivery date between 1 April 1991 and 31 December 1992. Note that the first official guidelines on prenatal alcohol consumption, mentioning that pregnant women should not drink more than 1–2 units of alcohol once or twice a week, were only issued by the UK Department of Health in 1995, after this cohort was born. Despite this, the US Surgeon General advised women not to drink in pregnancy as early as 1981 (Office of the US Surgeon General, 1981) and it is unlikely that UK women were completely insulated from this information. Approximately 85% of eligible mothers enrolled, leading to about 14,000 pregnancies (ALSPAC is a cohort; there is no systematic data collection on siblings).^9^ The Avon area has approximately 1 million inhabitants and is broadly representative of the UK as a whole, although slightly more affluent than the general population (Boyd *et al*., 2013; Fraser *et al*., 2013; see http://www.bristol.ac.uk/alspac for more a detailed description of the data).

Just over 12,000 children had at least one completed questionnaire. Our sample selection process is as follows. First, we exclude children whose mother or father is of non-white ethnic origin to reduce the risk of population stratification. Second, we select mothers for whom we observe both their and their children's genotype, leaving us with 5,531 observations.^10^ Third, we drop observations with missing data on all measures of prenatal alcohol exposure (*n* = 134), resulting in 5,397 observations. We further restrict the sample to children for whom we observe their academic achievement at least once. Depending on the measure of alcohol exposure and on the outcome of interest, the final sample includes between 1,922 and 4,088 mother–child matches.^11^

### Measures of Academic Achievement

We specify different measures of academic achievement. First, we use an entry assessment test, taken by all pupils about to start primary school (ages 4–5). Although there were no compulsory national assessment tests at this time, the Local Education Authorities covering the ALSPAC area used the same tests, which is available for 80% of (not privately owned) schools. In addition, we use four nationally set examinations taken at ages 7, 11, 14 and 16 (also known as the Key Stage 1 (KS1), Key Stage 2 (KS2), Key Stage 3 (KS3) and Key Stage 4 (KS4, or GCSE) examination, respectively). These measures of children's performance are objective and comparable across all children. Children's scores are obtained from the National Pupil Database, a census of all pupils in England within the state school system, which is matched into ALSPAC. For each of the Key Stage tests (1–4), we use an average score for the child's mandatory subjects, standardised on the full sample of children for whom data are available, with mean 0 and standard deviation 1.^12^

### >In Utero Exposure and the Genetic Marker

We use the binary genetic instrument *ADH1B*, comparing those with genotype GA or AA to those with genotype GG (A being the rare allele, where the effect is dominant; i.e. carrying one rare allele, GA, has a similar effect on alcohol consumption as carrying two, AA). Depending on the specification of interest, between 4.7% and 5.2% of our sample carry at least one rare allele.^13^

As discussed above, we would ideally use a direct measure of alcohol exposure *in utero*, such as foetal blood alcohol levels. As this is not available in the data, we proxy alcohol exposure *in utero* by maternal alcohol consumption during pregnancy. We start the analyses using a binary variable indicating whether the foetus was exposed to alcohol *in utero*. This equals one if the woman reports drinking any amount at any point during pregnancy and equals zero if the woman reports not drinking in the first, second, as well as third trimester, and reports not to have binged (i.e. has non-missing values for alcohol intake in each trimester).^14^ We then examine the effect of the dose of alcohol exposure, measured by the number of units consumed per week, averaged over the first, second and third trimester. In addition, we examine the pattern and duration of alcohol exposure. We proxy the pattern by investigating the effects of bingeing, defined by drinking the equivalent of two pints of beer, four glasses of wine or four pub measures of spirit in one day, measured in the second trimester. The duration is measured by a count variable ranging from 0 to 3, representing the number of trimesters during which the foetus was exposed to alcohol.

Several epidemiological studies distinguish between the effects of different types of beverages, noting increases in preterm births or decreases in birth weight primarily among beer drinkers (Kline *et al*., 1987). To investigate potential differences in the type of drink, we separately examine the effects of beer or wine consumption. This explores differences in (e.g.) wine consumption among those who report not consuming other alcoholic drinks. We do not use information on the consumption of spirits, as too few mothers report drinking spirits during pregnancy. The questionnaire explained that half a pint of ordinary strength beer, lager or cider, and a small glass of ordinary strength wine, contains one (UK) unit of alcohol (similar to 10 ml or 8 g of ethanol).

Note that all measures of alcohol exposure may be subject to substantial measurement error. First, the concentration of alcohol in different types of beers and wines varies considerably. Second, the size of a glass of wine in a bar or restaurant can vary anywhere between 125 and 250 ml. Third, these standard measures of 125 or 250 ml are only used in bars and restaurants; measures at home are likely to differ. Fourth, women may under-report their consumption during pregnancy (Gray and Henderson, 2006). Combining the measurement error with the imprecision and bias related to the reporting of alcohol consumption, this can lead to considerable underestimation of the amount of alcohol actually consumed (Stockwell *et al*., 2004), which may drive OLS estimates towards the null, though the IV may partially correct for this, assuming that the instrument is unrelated to the measurement error. We explore this assumption indirectly in subsection Descriptive Statistics, showing no systematic correlation between the instrument and a wide range of covariates.

### Covariates

Conditioning on covariates is not necessary to obtain unbiased estimates in Mendelian randomisation studies, as the covariates do not enter the assignment (randomisation) mechanism. In fact, it is unclear which covariates to include in a Mendelian randomisation study, as any characteristic is measured post-randomisation and – with that – may be affected by the instrument (von Hinke Kessler Scholder *et al*., 2011, 2013). For this reason, we do not control for covariates in our main analysis, though we discuss and report the estimates that adjust for a wide variety of different sets of covariates in subsection Robustness Checks and online Appendix D.

The exception, however, is that we include 10 ancestry-informative principal components to account for any remaining population stratification, and we control for the child's genotype. We include the latter for two reasons. First, when alcohol consumed by the mother crosses the placenta, the child's *ADH1B* may also start oxidising the ethanol (depending on whether the enzyme is expressed *in utero*). Second, the child's genotype is likely to be related to the child's alcohol consumption later in life, and may – through that – affect the child's academic achievement, although this is not likely for academic outcomes measured at younger ages. Including the child's *ADH1B* will account for these potential biases. However, the results are not sensitive to the inclusion or exclusion of the child's *ADH1B*.

### Descriptive Statistics

**Figure 2:**
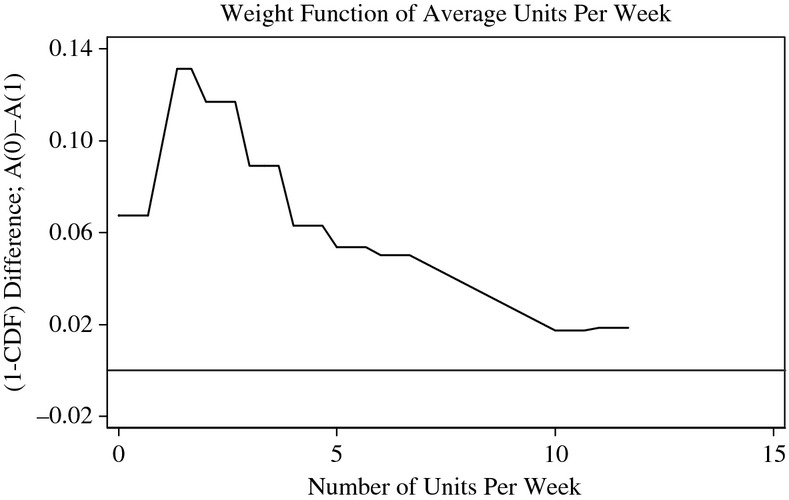
Weight Functions

As discussed in Section Methodological Framework, the IV estimate for the dose and duration of alcohol exposure is a weighted average of the unit causal response. Figure2 presents the weight function, plotting the differences (between those carrying no risk alleles and those carrying at least one risk allele for *ADH1B*) in the probability that alcohol intake is at or exceeds the level on the *x*-axis. This shows that those who carry no risk allele of *ADH1B* are between 2 and 13 percentage points more likely to drink, depending on the number of units examined. The intensity of the shift is highest around 2–3 units per week, declines thereafter, but remains positive throughout.

Table1 presents descriptive statistics of the average alcohol consumption during pregnancy. We show these for the full sample (column 1), as well as by genotype (columns 2 and 3). Panel (*a*) shows that, on average, 62.7% of the sample drank any alcohol during pregnancy, but this varies by genotype, with 63.3% of mothers who are homozygous for the common allele drinking alcohol, and 50.7% of those who carry at least one rare allele. Furthermore, we find that 17.0% of mothers who have two common alleles binged at least once in the second trimester, compared to 11.2% among those carrying at least one rare allele. Similarly, using the number of trimesters in which the mother drinks as a proxy for the duration or length of exposure, we find the average to be 0.99 for those carrying two common alleles, compared to 0.59 for those carrying at least one rare allele.

**Table 1:** Descriptive Statistics, Mean and Standard Deviation of Variables of Interest

	(1) Full sample		(2) Homozygous for the *ADH1B* common allele		(3) Carrying at least one *ADH1B* rare allele		(4) t-test
	Mean (SD)		Mean (SD)		Mean (SD)		p-value
*Panel* (*a*)*: any alcohol consumption*							
Any alcohol (binary)	0.627		0.633		0.507		0.001
	(0.484)		(0.482)		(0.501)		
*N*	4,201		3,990		211		
*Panel* (*b*)*: pattern and duration*							
Bingeing (trimester 2)	0.167		0.170		0.112		0.022
	(0.373)		(0.375)		(0.316)		
*N*	4,714		4,482		232		
Length of exposure	0.971		0.991		0.592		<0.001
	(1.165)		(1.172)		(0.963)		
*N*	2,880		2,733		147		
*Panel* (*c*)*: average alcohol consumption*							
No. of units p/w	1.503		1.549		0.646		<0.001
	(2.980)		(3.031)		(1.583)		
[min–max]	[0–35]		[0–35]		[0–11]		
*N*	2,781		2,639		142		
No. of units of wine	0.565		0.584		0.261		0.012
	(1.388)		(1.421)		(0.608)		
[min–max]	[0–17]		[0–17]		[0–3]		
*N*	2,116		1,991		125		
No. of units of beer	0.377		0.395		0.101		0.057
	(1.543)		(1.582)		(0.552)		
[min–max]	[0–35]		[0–35]		[0–5]		
*N*	1,803		1,697		106		

Standard deviations	Between	Within	Between	Within	Between	Within	
All alcohol	3.318	1.881	3.370	1.911	1.975	1.177	
Wine	1.796	1.143	1.818	1.153	1.285	0.930	
Beer	1.966	1.268	2.004	1.288	0.988	0.809	

Notes. The p-value is based on a test of equality between the mean for the homozygotes for the common allele and those carrying at least one rare allele. ‘Any alcohol’ is a binary variable indicating whether the foetus was exposed to any alcohol *in utero*. ‘Length of exposure’ ranges from zero to three trimesters. The average number of units of wine is calculated among women who either indicate to drink no beer, spirits or other alcoholic drinks, or who did not report their beer, spirit or other alcoholic consumption (i.e. have missing values for beer, spirit and other alcoholic drinks). Similarly, the average number of units of beer is calculated among women who either indicate to drink no wine, spirits or other alcoholic drinks, or who did not report their wine, spirit or other alcoholic consumption. Therefore, the sample sizes of the number of units of wine and beer do not add up to the total number of units. Indeed, some mothers may report drinking alcohol but do not define which drink they consumed.

The average number of units of alcohol per week is just over 1.5. However, there is much variation around this: the average number across pregnancy ranges from 0 to 35, with the variation in the number of beers being larger than that for wine. There are again large differences by maternal genotype, as shown in columns 2 and 3. Mothers who are homozygous for the common allele, for example, drink an average of 1.55 units a week. This is 0.65 units a week among those carrying at least one rare allele.

The second part of the table shows the between and within-standard deviations for the number of units of alcohol, wine and beer consumed for the full sample and the two genotypes separately. This shows that most of the variation lies between mothers, though there remains considerable variation within mothers. This suggests that mothers do alter their alcohol intake; it is not the case that mothers’ alcohol consumption remains stable over the course of their pregnancy. In other words, our results are not based on one particular group of mothers who do not change their behaviour during pregnancy.

To provide evidence on the validity of our IV approach, we exploit the richness of our data and correlate the instrument to an unusually extensive range of maternal and paternal prenatal background characteristics (we explore activities after birth – i.e. those that may be affected by child development – in subsection Parental Responsive Investments). This is presented in online Appendix B, showing the mean and standard deviation of a wide range of variables by the value of the instrument. With random assignment of genetic variants, there should be no systematic variation in covariates by genotype.

We start by testing covariates that are related to the (potential) alcohol intake of the mother's genetically related family. With each maternal allele having a 50% chance of being inherited by the child, children are more likely to carry the rare allele if their mother does. Similarly, we find that, among mothers who carry the rare allele, her mother and father are slightly less likely to have an alcohol problem. The mother's partner, however, is equally likely to drink during and after birth (at eight months) for mothers with or without the rare allele, suggesting that potential assortative mating based on alcohol consumption is not an important issue.

Our further extensive range of covariates includes:

- a set of ‘standard’ covariates;
- maternal tea/coffee/milk consumption;
- parental diet and nutrition,
- parental attitudes to breastfeeding and other parenting issues;
- religious beliefs;
- household and family characteristics;
- previous/current pregnancies and conditions during labour and delivery;
- mother's and partner's physical health, including a wide range of conditions measured both in the first and second trimester of pregnancy;
- mother's physical activity;
- measures of parental mental health;
- maternal use of medication in the first and second trimester as well as after pregnancy;
- parental substance use;
- mother's use of chemicals during pregnancy;
- the extent of social support available to the mother and partner; and
- neighbourhood characteristics.

All tests are reported in Table B1 in the online Appendix B, showing no systematic differences in the wide range of covariates by maternal genotype. We compare the number of correlations that are statistically significant with the number expected by chance if all variables were uncorrelated (excluding the characteristics of genetically related family). We find no greater association between the genetic variant and the covariates than what would be expected by chance (p = 0.32 at the 10% level, p = 0.46 at 5% and p = 0.48 at 1%), suggesting that the SNP is independent of behavioural or environmental factors that may affect the outcome of interest. Indeed, in the robustness checks in online Appendix D, we test the sensitivity of our analysis by controlling for these covariates in the IV specification, leaving our findings unaffected.

## Results

### OLS Results

Table2 presents the OLS estimates of the associations between prenatal alcohol exposure and child educational attainment, controlling for the ancestry-informative principal components and the child's *ADH1B*. Panel (*a*) reports the estimates for any alcohol exposure, showing an insignificant relationship with the different measures of child educational attainment, presented in the columns. Panel (*b*) shows a clear negative association between maternal binge drinking and educational achievement, whilst a longer exposure to alcohol is positively correlated with children's academic attainment.

**Table 2:** OLS Regressions of Child Academic Achievement on Maternal Prenatal Alcohol Consumption

	Entry assessment	KS1, age 7	KS2, age 11	KS3, age 14	KS4, age 16
*Panel* (*a*)*: any alcohol intake*					
Any alcohol intake	0.054	−0.037	0.026	0.026	−0.033
	(0.039)	(0.033)	(0.034)	(0.037)	(0.035)
*N*	2,614	3,319	3,132	2,872	3,201
*Panel* (*b*)*: pattern and duration*					
Bingeing	−0.107**	−0.210***	−0.159***	−0.225***	−0.235***
	(0.045)	(0.038)	(0.040)	(0.041)	(0.040)
*N*	3,238	4,088	3,868	3,572	3,955
Length of exposure	0.061***	0.028*	0.053***	0.046***	0.044***
	(0.019)	(0.015)	(0.015)	(0.017)	(0.016)
*N*	1,982	2,518	2,372	2,179	2,428
*Panel* (*c*)*: average alcohol intake*					
Average units of alcohol	0.010	−0.010*	−0.002	−0.006	−0.005
	(0.007)	(0.006)	(0.007)	(0.007)	(0.006)
*N*	1,922	2,433	2,293	2,106	2,345
Average units of wine	0.064***	0.033**	0.053***	0.052***	0.041***
	(0.016)	(0.014)	(0.013)	(0.017)	(0.014)
*N*	1,473	1,862	1,747	1,600	1,795
Average units of beer	−0.015	−0.044***	−0.039*	−0.061***	−0.049***
	(0.019)	(0.016)	(0.022)	(0.015)	(0.015)
*N*	1,275	1,569	1,475	1,381	1,521

Notes. The Table presents the correlations between academic achievement shown in the columns and the measures of alcohol exposure shown in the rows. All estimates come from separate regressions and control for ancestry-informative principal components and the child's *ADH1B*. Robust standard errors are in parentheses.

* p < 0.10

** p < 0.05

*** p < 0.01.

Examining the (average) number of units of alcohol in Panel (*c*) shows an ambiguous association; OLS coefficients are sometimes positive, sometimes negative, but most estimates cannot be distinguished from zero. In contrast, the Table shows strong positive correlations for exposure to wine, but negative associations for exposure to beer. Although this could reflect differential effects of wine and beer, it is more likely to simply reflect other characteristics of mothers who drink wine as opposed to beer during pregnancy.

Indeed, columns 1 and 2 in Table3 present the results from separate regressions of any alcohol and binge drinking, respectively, on the ‘standard’ covariates presented in online Appendix B, showing a strong socio-economic gradient in prenatal alcohol exposure. Mothers of higher socio-economic position are more likely to drink alcohol and less likely to binge, whereas length of exposure (column 3) is positively associated with socio-economic position. The positive gradient is stronger for wine consumption (column 5), than for mothers who drink beer or other alcoholic beverages (column 6): older, better educated, higher social class, employed mothers, and those with higher family income and a better educated, employed partner, are more likely to consume wine, whilst smoking, lower educated mothers with worse mental health are more likely to drink beer. This social gradient in alcohol consumption and the inverse gradient for binge drinking are consistent with those observed in other US and UK surveys.^15^

**Table 3:** The Correlation Between Alcohol Consumption and Background Characteristics

	(1) Any alcohol intake	(2) Binge drinking	(3) Length of exposure	(4) Average number of units of alcohol	(5) Average number of units of wine	(6) Average number of units of beer
*Covariates*						
Child's *ADH1B*	−0.116***	−0.026	−0.325***	−0.637***	−0.260**	−0.269**
	(0.038)	(0.024)	(0.090)	(0.203)	(0.132)	(0.108)
Girl	−0.031*	−0.006	−0.047	−0.121	0.019	−0.080
	(0.016)	(0.011)	(0.043)	(0.113)	(0.066)	(0.065)
Mother's age	0.008***	0.001	0.034***	0.068***	0.073***	0.016*
	(0.002)	(0.001)	(0.005)	(0.014)	(0.008)	(0.008)
Older siblings	0.032***	0.031***	0.041	0.214***	0.107**	0.114**
	(0.011)	(0.008)	(0.030)	(0.080)	(0.047)	(0.052)
Younger siblings	−0.006	0.021	0.062	0.152	0.250	0.097
	(0.038)	(0.028)	(0.107)	(0.298)	(0.200)	(0.194)
Father's education	0.035***	−0.024***	0.163***	0.169***	0.260***	−0.027
	(0.008)	(0.005)	(0.021)	(0.059)	(0.035)	(0.035)
Mother's education	0.026***	−0.039***	0.148***	0.043	0.249***	−0.141***
	(0.009)	(0.006)	(0.025)	(0.068)	(0.039)	(0.042)
Father's social class	0.027***	−0.021***	0.103***	0.098**	0.176***	−0.017
	(0.006)	(0.004)	(0.017)	(0.040)	(0.024)	(0.023)
Ln(income)	0.063***	−0.062***	0.359***	0.158	0.466***	−0.118
	(0.018)	(0.013)	(0.049)	(0.137)	(0.075)	(0.078)
Mother employed	0.056***	0.004	0.193***	0.128	0.214***	0.025
	(0.017)	(0.011)	(0.046)	(0.112)	(0.071)	(0.063)
Father employed	0.016	−0.065***	0.148**	−0.196	0.291***	−0.434**
	(0.027)	(0.019)	(0.070)	(0.241)	(0.096)	(0.189)
CCEI	0.005***	0.006***	0.007**	0.036***	0.008	0.020***
	(0.001)	(0.001)	(0.003)	(0.011)	(0.005)	(0.007)
EPDS	0.008***	0.009***	0.010**	0.058***	0.017**	0.031***
	(0.002)	(0.001)	(0.005)	(0.017)	(0.008)	(0.010)
Smoke (trimester 1)	0.084***	0.163***	0.108*	1.004***	0.010	0.775***
	(0.021)	(0.018)	(0.061)	(0.236)	(0.104)	(0.153)

Notes. The coefficient estimates are obtained from separate regressions of the alcohol exposure of interest (denoted in the columns) on each of the covariates in column 1. Robust standard errors are in parentheses.

* p < 0.10

** p < 0.05

*** p < 0.01.

### IV Results

Table4 presents the first-stage IV results, regressing prenatal alcohol exposure on the genetic instrument whilst controlling for the child's *ADH1B*. As expected, we find a negative correlation between maternal *ADH1B* and *in utero* alcohol exposure: mothers who carry at least one rare allele of *ADH1B* are less likely to drink any alcohol (column 1), less likely to binge (column 2), have a shorter duration of alcohol consumption (column 3) and drink fewer units of alcohol compared to those carrying two common alleles (columns 4–6). Hence, children born to these mothers have a reduced alcohol exposure during pregnancy. The F-statistic depends on the specification and sample size used, and is strongest when we consider the number of units of alcohol, ranging between 16 and 23. If we do not control for the child's genotype, this increases to 28–43, with similar point estimates and slightly smaller standard errors, suggesting that *ADH1B* predicts alcohol exposure well but that the inclusion of child *ADH1B* reduces its precision. The coefficients suggest that those who carry the rare allele are between 11 and 15 percentage points less likely to consume any alcohol during pregnancy. They drink between 0.77 and 0.86 units a week less compared to those not carrying the rare allele. The wine and beer-specific effects are smaller, though in the same direction. As discussed above, alcohol intake is only one of the three components through which the foetus may be exposed to alcohol and, hence, this is likely to be an underestimate of the effect of *ADH1B* on actual exposure.

**Table 4:** First-stage IV Results

	(1) Any alcohol intake	(2) Binge drinking	(3) Length of exposure	(4) Average number of units of alcohol	(5) Average number of units of wine	(6) Average number of units of beer
*Sample for entry assessment*						
*ADH1B*	−0.131**	−0.062**	−0.327***	−0.818***	−0.325**	−0.245***
	(0.051)	(0.028)	(0.113)	(0.189)	(0.128)	(0.063)
First-stage F-statistic	6.62	4.95	8.32	18.74	6.40	15.17
*N*	2,614	3,238	1,982	1,922	1,473	1,275
*Sample for KS1*						
*ADH1B*	−0.131***	−0.061**	−0.364***	−0.822***	−0.396***	−0.239***
	(0.046)	(0.025)	(0.108)	(0.203)	(0.115)	(0.051)
First-stage F-statistic	8.18	5.90	11.48	16.37	11.90	21.54
*N*	3,319	4,088	2,518	2,433	1,862	1,569
*Sample for KS2*						
*ADH1B*	−0.147***	−0.070***	−0.375***	−0.859***	−0.417***	−0.241***
	(0.048)	(0.025)	(0.106)	(0.189)	(0.111)	(0.049)
First-stage F-statistic	9.52	7.79	12.51	20.66	13.98	24.76
*N*	3,132	3,868	2,372	2,293	1,747	1,475
*Sample for KS3*						
*ADH1B*	−0.108**	−0.071***	−0.282**	−0.773***	−0.338***	−0.265***
	(0.050)	(0.026)	(0.118)	(0.208)	(0.131)	(0.058)
First-stage F-statistic	4.61	7.31	5.74	13.84	6.69	20.57
*N*	2,872	3,572	2,179	2,106	1,600	1,381
*Sample for KS4*						
*ADH1B*	−0.147***	−0.067***	−0.379***	−0.857***	−0.391***	−0.254***
	(0.047)	(0.025)	(0.105)	(0.180)	(0.107)	(0.049)
First-stage F-statistic	9.96	7.41	12.97	22.77	13.26	26.46
*N*	3,201	3,955	2,428	2,345	1,795	1,521

Notes. All estimates come from separate regressions and control for ancestry-informative principal components and the child's *ADH1B*. Robust standard errors are in parentheses.

* p < 0.10

** p < 0.05

*** p < 0.01.

The second-stage IV results are presented in Table5. To deal with potential weak instruments, we report the weak-instrument robust 95% confidence bounds, based on the Anderson Rubin statistic (Andrews *et al*., 2006). This shows consistent negative effects of any prenatal alcohol exposure, bingeing, the duration and the dose of alcohol exposure on all measures of child educational attainment, though due to the sometimes large standard errors, not all are significant. The magnitude of the estimates is considerable, though as we discuss above, we argue these are upper bounds of the causal effect of alcohol exposure.

**Table 5:** Second-stage IV Results

	(1) Entry assessment	(2) KS1, age 7	(3) KS2, age 11	(4) KS3, age 14	(5) KS4, age 16
*Panel* (*a*)*: any alcohol intake*					
Any alcohol intake	−0.685	−1.372*	−1.536*	−1.724	−1.557*
95% confidence intervals	[−4.45, 0.84]	[−4.85, −0.24]	[−4.59, −0.43]	[−18.6, −0.23]	[−4.57, −0.47]
*N*	2,614	3,319	3,132	2,872	3,201
*Panel* (*b*)*: pattern and duration*					
Bingeing	−1.782	−2.623*	−2.855*	−2.618*	−3.134*
95% confidence intervals	[−13.8, 1.51]	[−12.7, −0.46]	[−9.68, −0.84]	[−9.84, −0.58]	[−11.1, −1.06]
*N*	3,238	4,088	3,868	3,572	3,955
Length of exposure	−0.486	−0.591*	−0.693*	−0.665	−0.610*
95% confidence intervals	[−2.21, 0.13]	[−1.67, −0.14]	[−1.81, −0.20]	[−3.74, 0.01]	[−1.64, −0.13]
*N*	1,982	2,518	2,372	2,179	2,428
*Panel* (*c*)*: average alcohol intake*					
Average units of alcohol	−0.193	−0.245*	−0.298*	−0.232	−0.274*
95% confidence intervals	[−0.57, 0.06]	[−0.57, −0.05]	[−0.64, −0.08]	[−0.63, 0.02]	[−0.60, −0.06]
*N*	1,922	2,433	2,293	2,106	2,345
Average units of wine	−0.480	−0.554*	−0.657*	−0.520	−0.621*
95% confidence intervals	[−2.95, 0.21]	[−1.60, −0.10]	[−1.66, −0.17]	[−2.24, 0.14]	[−1.68, −0.10]
*N*	1,473	1,862	1,747	1,600	1,795
Average units of beer	−0.895	−1.061*	−1.462*	−1.176*	−1.105*
95% confidence intervals	[−2.53, 0.09]	[−2.39, −0.25]	[−2.96, −0.54]	[−2.54, −0.35]	[−2.40, −0.22]
*N*	1,275	1,569	1,475	1,381	1,521

Notes. All estimates come from separate regressions and control for ancestry-informative principal components and the child's *ADH1B*. Weak-instrument robust 95% confidence bounds in square brackets.

p < 0.05 using weak-instrument robust 95% confidence bounds.

Increasing the number of units of alcohol *in utero* lowers child academic attainment, with similar effect-sizes when examining the different educational outcomes. The estimates suggest that exposure to an additional unit of alcohol reduces academic achievement by up to 0.2–0.3 standard deviations. There is a slight suggestion that the negative effects of alcohol exposure increase as the child ages, with larger effects for the KS4 examination compared to the entry assessment or KS1 examinations, indicating possible accumulation of educational gaps and complementarity of educational achievement over time.

Examining the two types of alcoholic beverages, we find similar negative effects to the ‘average alcohol’ specification, though they are less well defined due to the smaller sample sizes, and larger due to the weaker first-stage association (and therefore smaller denominator in (1)). Note, however, that the instrument is not specific to wine or beer consumption, but to alcohol intake more generally. The estimates can therefore not be interpreted as the specific effect of wine or beer intake but rather indicate that the OLS associations, suggesting that wine improves and beer worsens child development, are likely to be biased due to unobserved confounding.

Although we argue that our IV estimates are an upper bound of the true causal effect, we are not the first to estimate such large effects, or to see a different association from the OLS after attempting to account for residual confounding. Indeed, Nilsson (2008) finds substantially large effects of prenatal alcohol exposure on human capital outcomes in Sweden. Similarly, Rosenzweig and Wolpin (1994) and Wüst (2010) obtain considerably larger negative effects in within-mother specifications compared to more ambiguous results in the OLS or GLS. Furthermore, it is consistent with the literature on the long-term effects of early-life conditions on later-life outcomes. This literature generally finds that foetal shocks have large impacts on later outcomes, including on test scores, educational attainment and other developmental outcomes (Currie, 2009; Almond and Currie, 2011). In addition and as discussed above, our measures of exposure are likely to be subject to considerable measurement error, which may drive OLS estimates towards the null. The IV, however, is not affected by this, resulting in larger estimates (in absolute value).

### Reduced Forms

Table6 presents the reduced form estimates from separate regressions of the test scores on the maternal genotype and regressions of the test scores on the child's genotype (Panel (*a*)); Panel (*b*) includes both genotypes simultaneously. All analyses control for the ancestry-informative principal components. Recall that exposure to alcohol *in utero* results from a combination of three components: maternal consumption, maternal metabolism and foetal metabolism. These analyses therefore shed light on whether the effect we find is likely to come via the combined consumption and metabolism through the mother, or via the foetal metabolism. We find a strong positive estimate for the maternal genotype, with much smaller and close-to-zero estimates for the child's genotype, suggesting that the alcohol effect runs through maternal intake and metabolism, rather than via the child metabolising its mother's alcohol.

**Table 6:** Reduced Form Estimates – Academic Achievement Regressed on Mother's and/or Offspring *ADH1B*

	(1) Entry assessment	(2) KS1, age 7	(3) KS2, age 11	(4) KS3, age 14	(5) KS4, age 16
*Panel* (*a*)*: separate regressions*					
Maternal *ADH1B* (rs1229984)	0.030	0.159***	0.180***	0.142*	0.214***
	(0.086)	(0.068)	(0.068)	(0.074)	(0.069)
*N*	2,564	3,255	3,067	2,812	3,138
Offspring *ADH1B* (rs1229984)	−0.146*	0.011	−0.007	−0.011	0.040
	(0.088)	(0.071)	(0.080)	(0.078)	(0.065)
*N*	2,564	3,255	3,067	2,812	3,138
*Panel* (*b*)*: including both genotypes simultaneously*					
Maternal *ADH1B* (rs1229984)	0.118	0.198***	0.239***	0.192***	0.250***
	(0.096)	(0.075)	(0.082)	(0.085)	(0.079)
Offspring *ADH1B* (rs1229984)	−0.202***	−0.082	−0.122	−0.103	−0.075
	(0.098)	(0.079)	(0.093)	(0.089)	(0.074)
*N*	2,564	3,255	3,067	2,812	3,138

Notes. All estimates come from separate regressions that control for 10 ancestry-informative principal components. Robust standard errors are in parentheses.

* p < 0.10

** p < 0.05

*** p < 0.01.

### The Prenatal Period

We are interested in the effect of prenatal alcohol exposure on child academic achievement. For mothers who carry a rare allele of *ADH1B*, however, their mother may also have been a carrier. As such, the mother's mother may have drunk less during her pregnancy, affecting the mother's cognitive abilities. This implies that we may not be able to attribute the entire observed effect to prenatal drinking by this generation of women alone, as there may also be indirect effects of drinking by the child's female ancestors. However, that does not provide evidence against a detrimental effect of prenatal alcohol exposure on child academic outcomes.

Furthermore, one may argue that our instrument does not solely explain prenatal drinking. In other words, mothers who carry the rare variant of *ADH1B* are likely to have had lower alcohol consumption throughout life. Hence, if the difference in alcohol exposure over the mother's lifetime changes her preferences or attitudes towards her child's education, the estimated effects are not necessarily solely due to prenatal alcohol consumption, but may partly reflect a more general alcohol intake.

Similarly, as alcohol consumption is correlated over the life cycle, our estimated negative effects may reflect the combined effects of alcohol consumption in different periods, rather than that specific to the prenatal period. For example, mothers who drink more may – perhaps because of that – spend less time with their children or pay less attention to their children's school performance. Or children whose mothers drink more may change their behaviour in response, affecting their outcomes.^16^ To examine these potential pathways, Table B1 (online Appendix B) explores whether *ADH1B* rare allele carriers have systematically different behaviours compared to those who carry two common alleles. We find no evidence of systematic differences by genotype.

Another possibility to explicitly examine the effects of prenatal alcohol exposure is by using SNPs that only affect exposure during pregnancy. Although the *ADH1B* effect is not specific to the pregnancy period, there is evidence that *ADH1B* is a stronger predictor of alcohol intake and quitting during pregnancy, compared to that in other periods (Jacobson *et al*., 2006; Zuccolo *et al*., 2009; Wehby and von Hinke Kessler Scholder, 2013).^17^ Hence, we can examine the effect of quitting during pregnancy. If prenatal alcohol exposure negatively affects child academic achievement, we would expect to find a positive effect on child academic attainment for those children whose mother's *ADH1B* induced them to quit drinking during pregnancy. To investigate this, we restrict the sample to women who drank prior to pregnancy and define quitters as those reporting not drinking at any point during pregnancy.^18^ The findings (available from the authors upon request) show consistent positive effects of quitting during pregnancy on child educational attainment, with estimates of very similar (absolute) magnitude to those in Panel (*a*) of Table5. As above, the results are likely to be an overestimate due to not being able to measure actual exposure to alcohol. Nevertheless, the direction of effect is as expected. Hence, although we are not able to fully deal with the specificity of the prenatal period, our results are at least suggestive that alcohol exposure during the intrauterine period affects the foetus.

### Parental Responsive Investments

The large estimates of the effect of prenatal alcohol exposure on child educational attainment call for an investigation into the potentially differential investments that parents make in response to their child's development. The literature on parental responsive investments tends to explore whether they reinforce or compensate for initial endowment differences (for a recent overview of the literature, see Almond and Mazumder, 2013). Understanding these responses is of broad interest and can provide interesting insights into parental responsive investment behaviours.

To explore this in detail, we estimate IV regressions to examine whether alcohol exposure *in utero* leads to differential parental responses, considering a wide range of post-birth characteristics and behaviours that parents have control over. These include:

- child diet and nutrition;
- immunisations and other health treatments (such as fluoride treatment and the use of vitamins);
- interactions between the parents and child;
- doctor and dentist visits;
- parenting and teaching scores of both the mother and her partner;
- time use;
- maternal worries and concerns about her child;
- a set of post-birth household characteristics;
- the use of child care; and
- the level of social support and social network available to the mother and her partner.

In addition, many of these variables are observed multiple times after birth, allowing us to also explore whether any differences are systematic over time.

The results are presented in Table C1 of online Appendix C. These show some significant effects of alcohol exposure *in utero*. For example, consuming alcohol during pregnancy increases (decreases) the likelihood of having given the baby formula (a herbal drink) at age six months. However, we find little evidence of any systematic patterns in the data that would suggest that prenatal alcohol consumption leads to differential parental choices and behaviours. For example, parents are more likely (at age six months) to change nappies at night of babies exposed to alcohol *in utero*, but there is no difference in night-time nappy changing at age four weeks. Similarly, we find that parents of children who are exposed to alcohol *in utero* are less likely to take their child to the dentist or use a toothbrush/toothpaste at 38 months but they are more likely to have a doctor visit at 18 and 30 months. The only finding that is consistent over time is that exposure to alcohol increases the likelihood that babies are regularly looked after by their grandparents at age 15, 24 and 38 months. Considering the wide range of parental choices explored, however, there seems to be little evidence of any systematic differences in parental responsive investments for children exposed to alcohol *in utero* compared to those not exposed, suggesting that most of the effect we find from prenatal alcohol consumption on academic achievement in foetal in origin.

### Subgroup Analysis

To examine whether the effects of alcohol exposure are different for different groups of children, we distinguish between child's gender, mother's age at birth, partner's social class at birth, maternal education and family income. The results are reported in Table7. Consistent with Nilsson (2008), the estimates are slightly larger for children of lower educated and lower income mothers. In contrast to previous findings that show boys to be more vulnerable to alcohol exposure *in utero* than girls (Nilsson, 2008; Barreca and Page, 2012), however, we find no clear patterns by gender or social class.

**Table 7:** Subgroup Analysis, Number of Alcoholic Units

	(1) Entry assessment	(2) KS1, age 7	(3) KS2, age 11	(4) KS3, age 14	(5) KS4, age 16
*By gender*					
Boys	−0.167	−0.278**	−0.217	−0.240*	−0.390**
	(0.170)	(0.128)	(0.133)	(0.141)	(0.168)
First-stage F-statistic	12.832	15.681	15.618	12.007	15.307
*N*	1,000	1,239	1,151	1,060	1,188
Girls	−0.182	−0.185	−0.438	−0.180	−0.087
	(0.212)	(0.207)	(0.274)	(0.310)	(0.192)
First-stage F-statistic	7.838	4.200	7.533	3.740	9.415
*N*	922	1,194	1,142	1,046	1,157
*By mother's age at birth*					
Mothers aged 27 or less	0.150	−0.233	−0.420	−0.412	−0.174
	(0.239)	(0.227)	(0.307)	(0.291)	(0.221)
First-stage F-statistic	5.362	6.317	5.141	4.864	7.030
*N*	789	962	909	867	928
Mothers aged over 27	−0.424**	−0.265**	−0.261**	−0.158	−0.326**
	(0.185)	(0.126)	(0.129)	(0.162)	(0.141)
First-stage F-statistic	11.197	9.545	13.909	7.712	13.849
*N*	1,133	1,471	1,384	1,239	1,417
*By social class*					
Low social class	−0.221	−0.255*	−0.440**	−0.305*	−0.293*
	(0.213)	(0.144)	(0.204)	(0.172)	(0.176)
First-stage F-statistic	7.232	9.245	10.037	7.951	9.939
*N*	817	977	941	889	952
High social class	−0.262	−0.303*	−0.184	−0.184	−0.258
	(0.237)	(0.180)	(0.164)	(0.240)	(0.178)
First-stage F-statistic	8.050	6.593	9.273	5.496	11.589
*N*	1,010	1,346	1,254	1,118	1,287
*By maternal educational level*					
Low education	−0.188	−0.279	−0.507	−0.232	−0.433*
	(0.313)	(0.209)	(0.310)	(0.230)	(0.257)
First-stage F-statistic	3.422	5.770	4.878	4.241	6.395
*N*	1,231	1,478	1,414	1,353	1,444
High education	−0.156	−0.140	−0.060	−0.106	−0.051
	(0.126)	(0.105)	(0.086)	(0.114)	(0.100)
First-stage F-statistic	20.346	10.947	20.855	11.266	21.018
*N*	688	952	876	750	898
*By income*					
Low income (less than median)	−0.404	−0.319	−0.405	−0.258	−0.321
	(0.272)	(0.195)	(0.260)	(0.271)	(0.212)
First-stage F-statistic	6.580	8.471	7.814	4.602	8.437
*N*	853	1,027	978	939	1,005
High income (more than median)	0.007	−0.081	−0.104	−0.087	−0.061
	(0.199)	(0.131)	(0.110)	(0.139)	(0.136)
First-stage F-statistic	7.161	5.263	8.165	4.775	9.427
*N*	820	1,090	1,020	883	1,040

Notes. All estimates come from separate regressions where the treatment of interest is the number of alcoholic units consumed. All analyses control for ancestry-informative principal components and the child's *ADH1B*. Robust standard errors are in parentheses. Low social class indicates non-skilled, semi-skilled or skilled manual occupations; High social class indicates skilled non-manual, managerial or professional occupations. Low education denotes O-level or less, high education indicates A-level or university degree.

* p < 0.10

** p < 0.05

***p < 0.01.

### Low-to-moderate Drinking

**Figure 3:**
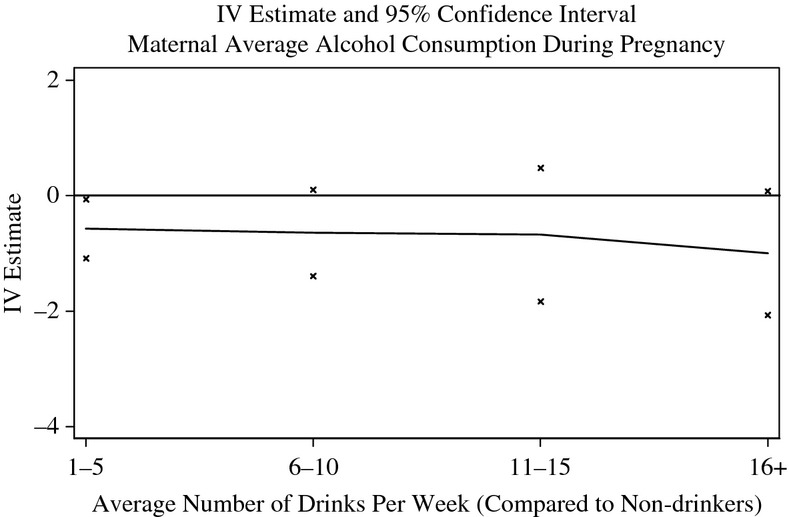
The Effect of Low-to-moderate and Heavy Drinking on the Child's KS1 Score*Note*. The 95% confidence intervals are presented as two points above and below the estimate.

The UK Department of Health suggests that, if women choose to drink during pregnancy, they should not exceed 1–2 units once or twice a week, as ‘at this low level, there is no evidence of any harm to the unborn baby’. If there truly are non-linearities in the effects of alcohol exposure *in utero*, we cannot directly investigate this with only one instrument. To shed some more light on this, however, Figure3 plots the IV point estimates for mothers drinking 1–5, 6–10, 11–15 and 16 or more units a week, comparing each of them to mothers who do not drink. This shows that all estimates are negative, including the indicators for low-to-moderate consumption, though not all are very precisely estimated. Nevertheless, this does suggest that low-to-moderate alcohol exposure also harms the foetus.

### The Timing of Exposure

For policy purposes, whether there is any differential effect in the timing of exposure to alcohol *in utero* is of substantial interest. Although we observe the number of drinks in each trimester and we can run the analyses separately by trimester, the interpretation of the estimates is limited by the fact that the instrument is not specific to a particular trimester. In other words, as the reduced form (the numerator in (1)) is similar in all analysis for a specific outcome variable (apart from differences due to the sample size), changes in the IV estimates are mainly driven by differences in the first stage (the denominator in (1)). Indeed, unsurprisingly, our results (available upon request) suggest that the estimates are similar throughout pregnancy.

### Robustness Checks

We perform a range of checks to verify that our results are robust to different specifications, shown in online Appendix D. We present the estimates of the average number of units per week during her pregnancy on KS1 scores, though the findings are robust to the use of the entry assessment test, or later KS examinations. The different model specifications control for different sets of covariates. We start by controlling for a set of alcohol-related variables (Panel (*a*)): specification 1 repeats the KS1 results from Table5 for comparison; specification 2 includes an indicator for maternal smoking during pregnancy; specification 3 does not include the child's *ADH1B* (i.e. only including the principal components); specification 4 includes (binary) indicators for maternal post-natal alcohol intake when the child was 8, 21, 33 and 47 months old; specification 5 includes binary indicators for the child's own alcohol consumption at 157, 166 and 185 months; specification 6 accounts for the mother's partner's alcohol consumption in the second trimester, the partner's alcohol intake and bingeing at eight months and whether the mother's parents ever had an alcohol problem.

We next run multiple IV analyses, each time controlling for the different sets of characteristics and behaviour listed in Table B1 of online Appendix B. For these analyses, we only control for the mother's characteristics, as sample sizes fall substantially when controlling for partner's characteristics due to missing values. However, as most variables relate to the mother, this still controls for an extensive set of covariates that are generally not observed in survey data. Panel (*b*) shows that the use of different sets of control variables leads to different sample sizes due to missing values on some covariates. However, our results are very robust, with coefficients of similar magnitudes in all specifications.

## Discussion and Conclusion

This article examines the effect of alcohol exposure *in utero* on child academic achievement. Simple correlations between alcohol exposure and child academic achievement show somewhat ambiguous results, with exposure to wine having a positive association but exposure to beer being negative. Binge drinking is bad for the child but a longer duration of exposure is positively associated with the child's academic performance. However, we present clear evidence of the endogeneity of alcohol intake, showing a strong social gradient in maternal alcohol consumption, with mothers of higher socio-economic status more likely to drink and, in particular, drink wine. In contrast, beer consumption is associated with lower education and worse mental health. To deal with the confounding, we use a genetic variant in the alcohol metabolism gene *ADH1B* as an instrument for alcohol exposure, and show that – in contrast to alcohol consumption – the genetic instrument is unrelated to potential confounders, examining an unusually wide range of maternal and paternal characteristics and behaviours. We include a detailed discussion of the IV assumptions that are required to estimate the causal effect of alcohol exposure. In stark contrast to the OLS, our IV estimates show large negative effects of prenatal alcohol exposure on child educational achievement, which are robust to a large set of model specifications. In addition, the reduced form regressions show that the effects are solely driven by the maternal genotype, with no impact of the child's genotype. Yet, despite the large negative effects, we find little evidence of differential parental responses to child development, exploring a wide range of parental post-natal investments and behaviour.

Our estimates are local average treatment effects (LATE), capturing the effect on children whose mother was induced by her genotype to reduce her alcohol intake. Although we obviously cannot alter individuals' genotypes, we believe that our estimates remain policy relevant. As argued in Imbens (2010), if randomised experiments are unethical or infeasible, credible evaluations can be based on instrumental variable strategies. Although they are second best to randomised experiments, as they rely on additional assumptions and have less external validity, they are often all we have. The relatively small number of studies attempting to deal with the endogeneity of prenatal alcohol exposure indeed suggests that it is particularly difficult due to unobserved residual confounding. Using different methodological approaches, these studies find negative effects of prenatal alcohol exposure on child development (Rosenzweig and Wolpin, 1994; Nilsson, 2008; Fertig and Watson, 2009; Wüst, 2010; Zhang, 2010). There is no evidence *a priori* to suggest that different sources of variation in alcohol exposure lead to different effects of exposure on academic achievement. In addition, if there is a biological effect of alcohol exposure (damaging the developing brain), any reduction in exposure should improve child outcomes. Hence, despite estimating a LATE, we believe that our estimates have some external validity and are relevant to policy.

Although the mothers in our sample were pregnant before the official UK guidelines on prenatal alcohol consumption were released, we believe our results are still likely to be relevant in today's context for three reasons. First, the US Surgeon General advised women not to drink during pregnancy as early as 1981 and it is unlikely that UK women were completely insulated from this information. Second, with the UK's most recent guidelines on alcohol consumption during pregnancy being very similar to their first guidelines, we assume that differences in the information available between the early 1990s and today are modest. Third, it is unlikely that the biological effects of alcohol exposure on child development have changed over time, suggesting that the results are also relevant for today's society.

Although we argue that our estimates may be an upper bound, they are very robust to different model specifications. In addition, we are not the first to find such large effects: the few papers that attempt to deal with unobserved confounding in alcohol exposure also find large negative effects on child development (Nilsson, 2008; Wüst, 2010; Zhang, 2010).

Nevertheless, the article has several limitations. First, we are not able to deal fully with the specificity of the prenatal period. Second, we cannot make any strong statements about the specific effects of low-to-moderate *versus* excessive prenatal alcohol intake, though the analyses suggest that both negatively affect child academic attainment. Third, although the results suggest that the effects are similar for alcohol intake throughout pregnancy, we cannot rule out differential effects of the timing of exposure. Fourth, as with any other IV analyses, the validity of independence and exclusion will never be known with complete certainty. However, the well-known mechanism of *ADH1B*, its location on the chromosome, the literature search on the effects of *ADH1B* and our extensive tests examining the distribution of child and family characteristics by genotype all suggest that the SNP is independent of behavioural or environmental factors that may affect the outcome of interest.

Hence, by examining the link between prenatal alcohol exposure and child educational outcomes, this article contributes to the economic literature on the long-term effects of the early environment on later child outcomes (Almond, 2006; van den Berg *et al*., 2006; Currie, 2009; Almond and Currie, 2011; Almond and Mazumder, 2011), on potential differential investments by parents in response to child development (Almond and Mazumder, 2013) and on identifying critical and sensitive periods of parental investments *per se* (Cunha and Heckman, 2007). We also provide advice to policy makers, showing that low-to-moderate alcohol exposure *in utero* may have similar negative effects on the foetus that may be carried into childhood and adolescence. In addition, as it is unethical to design a randomised controlled trial to study foetal alcohol exposure, we show that quasi-experimental designs such as Mendelian randomisation can provide powerful alternatives for causal inference.

## Appendix

### Appendix A. Brief Introduction to Genetics

Each cell in the human body contains a nucleus in which most DNA (99.9995%) is located. DNA forms structures called chromosomes, where each chromosome contains a single continuous piece of DNA. All cells in the human body apart from gametes (i.e. germ cells) contain 46 chromosomes, organised into 23 chromosome pairs: one copy of chromosome 1–22 from each parent, plus an X-chromosome from the mother and either an X or a Y chromosome from the father.

Sites within DNA that vary between people are called polymorphisms. The most commonly studied form of polymorphism is a single nucleotide polymorphism (SNP): a single base-pair variation in a particular location on the DNA sequence. As chromosomes come in pairs, humans have two base-pairs at each location (locus). Where there are two or more forms of DNA at a specific locus, these different forms are called alleles. The term genotype is used to describe the specific set of alleles inherited at a particular location on the chromosome. For example, where individuals can have one of two alleles on each chromosome at the rs1229984 locus (A or G), this will result in three genotypes: they can be homozygous for the common allele (having two of the same common/most prevalent alleles: GG), heterozygous (AG) and homozygous for the rare allele (AA).

## Notes

1 Including, for example, *LAC1* (rs4988235), *CETP* (rs708272), *TNF-α* (rs1800629), *GPX4* (rs1007), *MTHFR* (rs1801133), *FTO* (rs9939609), as well as the variant used here (*ADH1B*, rs1229984).

2 Such as the British Women Heart and Health Study, the Young Finns Study, the Copenhagen General Population Study, the Avon Longitudinal Study of Parents and Children, as well as different case–control samples.

3 These include more ‘medical’ characteristics (e.g. pulse, lung function, vitamin levels, haemoglobin, fasting insulin, fasting glucose, fibrinogen, C-reactive protein, plasma viscosity, etc.) as well as socio-economic or behavioural characteristics (e.g. area deprivation, SES, types of foods/drinks consumed, time use, walking speed, educational level, age when parents died, housing characteristics, nurse estimation of life expectancy etc.)

4 For example, Bhatti *et al*. (2005) explore differences in polymorphism frequencies by willingness to participate in studies. They examine three studies with different recruitment designs and different participation incentives. Conditional on having provided blood or saliva samples, they investigate whether genotype frequencies differ by the timing of non-response to questionnaires (early, late and never responders), finding no systematic correlations. Davey Smith *et al*. (2008) estimate pairwise correlations between non-genetic and genetic variables and compare the number of correlations that are statistically significant with the number expected by chance if all variables were uncorrelated. They show significant correlations between behavioural, socio-economic and physiological factors, with 45% of the 4,560 pairwise correlations being significant at the 1% level. In contrast, genetic variants show no greater association with each other, nor with the behavioural, socio-economic and physiological factors than what would be expected by chance. Consistent with these findings, the allele frequencies in British blood donors have been shown to be virtually identical to those in the British 1958 cohort study (WTCCC, 2007). The former are clearly a highly selected sample in the population, whereas the latter includes a nationally representative sample of all children born in one week in Britain. Taken together, this suggests that genetic variants are generally unrelated to potential confounders.

5 Other studies in the epidemiology literature include Lewis *et al*. (2012) and Zuccolo *et al*. (2013).

6 Although not the main research question in their study, Rosenzweig and Wolpin (1994) also do not find any effects of maternal prenatal alcohol consumption on child test scores in their GLS estimation, but the estimates become negative when using a within-mother specification.

7 Ethanol is also known as pure alcohol or drinking alcohol. It is the type of alcohol found in alcoholic beverages.

8 The second step in the metabolism of ethanol is mainly driven by aldehyde dehydrogenase enzymes. Some individuals carry a polymorphism in the ALDH2 gene which encodes an enzyme that is unable to clear acetaldehyde, leading to severe symptoms of facial flushing, increased heart rate and nausea, causing these individuals to abstain from alcohol or drink very little. This *ALDH2* variant has been used in Mendelian randomisation studies to explore the causal effects of alcohol consumption on blood pressure (Chen *et al*., 2008), drug use and anti-social behaviour (Irons *et al*., 2007) and upper aerodigestive and stomach cancers (Zhang *et al*., 2007; Hashibe *et al*., 2008). However, its relevance is limited to East-Asian populations as the variant is absent in populations of European ancestry.

9 Of the 14,676 foetuses with a known birth outcome, 14,062 were live births and 13,988 were alive at one year. As we do not observe the genotype of mothers whose children did not survive, we cannot directly explore whether alcohol exposure *in utero* affects survival rates. However, if the genotype affects the survival of foetuses, it would not be in Hardy–Weinberg equilibrium (which states that allele and genotype frequencies in a population remain constant from generation to generation in the absence of other evolutionary influences, such as non-random mating and selection). We checked this and *ADH1B* is in equilibrium.

10 For our sample of mother–child pairs, we observe a total of 7,088 maternal genotypes and 8,886 child genotypes at rs1229984. As we require both genotypes in the analyses, we can only use those observations for which we observe the two: *n* = 5,513. It is unlikely that missing genotype data introduce selection bias. First, empirical evidence on other data suggests that genotype frequencies are the same for general population *versus* selected samples (e.g. the British 1958 birth cohort *versus* British blood donors from the Wellcome Trust Case Control Consortium; see WTCCC, 2007). Second, empirical evidence shows that genotype frequencies do not differ by the timing of non-response to questionnaires (Bhatti *et al*., 2005). And third, we examine potential bias in our data due to missing genotypes by investigating whether, conditional on observing genetic information, the probability of being in the final sample differs by maternal and child genotypes. In other words, we test whether the genotype frequencies for mother and child differ, comparing the sample where we observe both genotypes (i.e. our estimation sample) to the sample where we only observe just the mother's or just the child's genotype. We find no evidence that mother or child genotype frequencies differ between the two samples (p = 0.12 and p = 0.58, respectively), suggesting that the missingness is unrelated to the instrument.

11 The low sample sizes are mainly driven by third trimester alcohol intake for which an additional 35% (of the maximum of 5,397 observations) is missing. Lower educated women are more likely to be missing in the third trimester. However, this does not affect our results, which are robust the use of different samples. More generally, Boyd *et al*. (2013) and Fraser *et al*. (2013) show that the lower socio-economic groups are more likely to attrite.

12 For KS1, this is an average of the child's reading, writing, spelling and mathematics scores; KS2 includes reading, writing, science and mathematics. For KS3 and KS4, the final score is an average of the child's English, mathematics and science.

13 With our sample sizes, this corresponds to 106–267 mothers (presented in Table1); a relatively low number.

14 Alcohol consumption in the first, second and third trimesters are obtained from questionnaires at 8, 18 and 32 weeks' gestation, respectively. Note that the first trimester questionnaire was only sent out to mothers who enrolled before 14 weeks' gestation; this is almost half of all mothers in our data. The other half were asked about their first trimester alcohol consumption at a later date. For ease of description and discussion, however, we refer to this as the week eight, or first trimester, questionnaire. In the first and third trimester, women were directly asked to report the number of alcoholic units consumed per week. In the second trimester, women were asked to report the number of units of beer, wine, spirits and other alcohol per week, which we sum to obtain the total number of units.

15 Although the majority of these explore the social gradient in alcohol consumption in general; see e.g. Cutler and Lleras-Muney (2010) using the National Health and Interview Survey, the Health and Retirement Study, the National Longitudinal Survey of Youth 1979 and the National Child Development Study); others explore alcohol intake during or just after pregnancy; e.g. Bartley *et al*. (2005) and Dezateux *et al*. (2005) using the UK Millennium Cohort Study.

16 Although we observe some variables for maternal alcohol intake post-pregnancy, these are categorical variables and therefore the magnitude of any OLS or IV estimates are not comparable to those reported above.

17 This could suggest an interaction between *ADH1B* and the environment, the latter being pregnancy. With women facing similar environments during pregnancy, those who carry *ADH1B* may find it easier to quit. Alternatively, it could mean that *ADH1B* causes other (physical) changes in pregnancy that lead these women to quit drinking. Although there is no evidence of the latter, and we find no evidence of this in Table B1, it could violate the IV assumption depending on such other effects of the variant.

18 In other words, this definition is perfectly negatively correlated with our definition of ‘any alcohol’ exposure but the analysis is conditional on the sample of women who drank before pregnancy.

## Notes

**Appendix B.**Tests of Independence. **Appendix C.**Parental Responsive Investments. **Appendix D.** Robustness Checks.
