# Supplementary material for: Alcohol Exposure In Utero and Child Academic Achievement*
Source: Econ J (London). 2014 May 23;124(576):634–67. doi: 10.1111/ecoj.12144 (PMC4243528; doi:10.1111/ecoj.12144)
Supplement: Supplementary file 1 — Appendix B.Tests of Independence. Appendix C.Parental Responsive Investments. Appendix D. Robustness Checks. [file ecoj0124-0634-sd1.pdf]

The Economic Journal, Doi: 10.1111/ecoj.12144 © 2014 The Author(s). The Economic Journal published by John Wiley & Sons Ltd on behalf of Royal Economic Society. Published by John Wiley & Sons, 9600 Garsington Road, Oxford OX4 2DQ, UK and 350 Main Street, Malden, MA 02148, USA.

Technical Appendix to

ALCOHOL EXPOSURE *IN UTERO* AND CHILD ACADEMIC ACHIEVEMENT

Stephanie von Hinke Kessler Scholder, George L. Wehby, Sarah Lewis and Luisa Zuccolo

ECONOMIC JOURNAL, doi: 10.1111/ecoj.12144

Appendix B. Tests of Independence

To provide evidence on the validity of our IV approach, Table B1 presents descriptives of the covariates presented in the first column by genotype. Column 3 shows the p-value of a test whether the mean among those homozygous for the common allele (column 1) equals the mean among those carrying at least one rare allele (column 2). With random assignment of genetic variants, there should be no systematic variation in covariates by genotype. Table B1 shows this for a wide range of maternal and paternal prenatal background characteristics and behaviour (we investigate activities after birth – i.e. those that may be affected by child development, exploring potential parental responsive investments – in subsection 4.5).

We compare the number of correlations that are statistically significant with the number expected by chance if all variables were uncorrelated (excluding the first set of covariates, which concern genetically related family members). We find no greater association between the genetic variant and covariates than what would be expected by chance ($p = 0.32$ at the 10% level, $p = 0.46$ at 5% and $p = 0.48$ at 1%), suggesting that the SNP is independent of behavioural or environmental factors that may affect the outcome of interest.

Table B1

Descriptive Statistics: Mean and Standard Deviation of Covariates

	(1) Mother is homozygous for the common allele at rs1229984		(2) Mother carries at least one rare allele at rs1229984		(3) t-test
	Mean	SD	Mean	SD	p-value
Alcohol-related covariates of mother's family					
Child's *ADH1B* (rs1229984)	0.029	0.168	0.471	0.500	<0.001
Mother's mother has alcohol problem	0.022	0.147	0.008	0.091	0.125
Mother's father has alcohol problem	0.054	0.226	0.034	0.181	0.094
Alcohol-related covariates of mother's partner					
Partner drinks any alcohol (at 18 weeks' gestation)	0.705	0.456	0.728	0.446	0.436
Partners drinks any alcohol (at eight months)	0.749	0.434	0.702	0.457	0.573
Frequency of partner drinks > 4 units (eight months; 0 = never, 5 = daily)	2.088	1.390	1.966	1.558	0.315

Table B1

(*Continued*)

	(1) Mother is homozygous for the common allele at rs1229984		(2) Mother carries at least one rare allele at rs1229984		(3) t-test
	Mean	SD	Mean	SD	p-value
*'Standard' covariates**					
Girl	0.482	0.500	0.505	0.501	0.449
Child's age at KS1 (in months)	88.727	3.735	88.302	3.673	0.096
Mother's age at child's birth (in years)	28.543	4.666	28.651	4.555	0.709
Older siblings (0, 1 or 2+)	0.726	0.748	0.727	0.746	0.972
Younger siblings (0, 1 or 2+)	0.048	0.220	0.075	0.279	0.068
Father's education: O-level	0.309	0.462	0.293	0.456	0.582
Father's education: A-level	0.275	0.447	0.293	0.456	0.533
Father's education: university degree	0.190	0.392	0.202	0.402	0.646
Mother's education: O-level	0.444	0.497	0.451	0.499	0.809
Mother's education: A-level	0.233	0.423	0.257	0.438	0.366
Mother's education: university degree	0.146	0.353	0.153	0.361	0.749
Social class: semi-skilled	0.098	0.297	0.070	0.255	0.145
Social class: skilled manual	0.301	0.459	0.332	0.472	0.304
Social class: skilled non-manual	0.114	0.318	0.082	0.275	0.119
Social class: managerial/technical	0.349	0.477	0.361	0.481	0.718
Social class: professional	0.114	0.317	0.119	0.324	0.798
Ln (income)	5.331	0.479	5.352	0.452	0.510
Mother employed	0.499	0.500	0.444	0.498	0.103
Father employed	0.873	0.333	0.897	0.305	0.274
CCEI (score ranging from 0 to 44)	13.009	7.485	12.943	7.222	0.894
EPDS (score ranging from 0 to 23)	6.629	4.723	6.440	4.760	0.538
Smoking (first trimester)	0.172	0.377	0.136	0.343	0.131
Mother's tea, coffee and milk, eight weeks' gestation					
Drink tea	0.799	0.401	0.749	0.434	0.050
Drink decaf tea	0.035	0.184	0.034	0.182	0.951
Drink coffee	0.511	0.500	0.492	0.501	0.556
Drink decaf coffee	0.157	0.364	0.130	0.337	0.249
Drink cola	0.347	0.476	0.391	0.489	0.149
Drink decaf cola	0.090	0.287	0.080	0.272	0.576
Drink milk	0.586	0.493	0.604	0.490	0.561
Parental diet and nutrition†					
Mother eats sausages/burgers	0.608	0.488	0.584	0.494	0.448
Mother eats pies or pastries	0.550	0.498	0.506	0.501	0.169
Mother eats meat	0.913	0.281	0.898	0.303	0.400
Mother eats poultry	0.906	0.292	0.898	0.303	0.665
Mother eats offal	0.093	0.290	0.075	0.263	0.320
Mother eats white fish	0.823	0.382	0.843	0.364	0.413
Mother eats oily fish	0.593	0.491	0.612	0.488	0.543
Mother eats shellfish	0.193	0.394	0.196	0.398	0.893
Mother eats eggs or Quiche	0.861	0.346	0.859	0.349	0.908
Mother eats cheese	0.945	0.227	0.953	0.212	0.600
Mother eats pizza	0.571	0.495	0.643	0.480	0.024
Mother eats chips	0.819	0.385	0.776	0.417	0.085
Mother eats roast potatoes	0.697	0.460	0.702	0.458	0.858
Mother eats boiled or baked potatoes	0.975	0.156	0.980	0.139	0.587
Mother eats boiled rice	0.767	0.423	0.788	0.409	0.432

© 2014 The Author(s).
The Economic Journal published by John Wiley & Sons Ltd on behalf of Royal Economic Society.

Table B1

(*Continued*)

	(1) Mother is homozygous for the common allele at rs1229984		(2) Mother carries at least one rare allele at rs1229984		(3) t-test
	Mean	SD	Mean	SD	p-value
Mother eats pasta	0.811	0.392	0.816	0.389	0.843
Mother eats crisps	0.792	0.406	0.769	0.423	0.372
Mother eats fried food	0.486	0.500	0.427	0.496	0.067
Mother eats baked beans	0.853	0.354	0.859	0.349	0.803
Mother eats peas or corn	0.933	0.251	0.941	0.236	0.592
Mother eats cabbage	0.904	0.294	0.898	0.303	0.745
Mother eats other green vegetables	0.938	0.242	0.925	0.263	0.429
Mother eats carrots	0.929	0.257	0.929	0.257	0.970
Mother eats root vegetables (not carrots)	0.629	0.483	0.580	0.494	0.120
Mother eats salad	0.916	0.277	0.933	0.250	0.339
Mother eats fresh fruit	0.979	0.143	0.988	0.108	0.320
Mother drinks tinned juice	0.188	0.391	0.122	0.327	0.008
Mother drinks pure non-tinned juice	0.788	0.408	0.831	0.375	0.101
Mother eats pudding	0.759	0.428	0.780	0.415	0.432
Mother eats oat cereals	0.559	0.497	0.537	0.500	0.491
Mother eats bran cereals	0.686	0.464	0.722	0.449	0.234
Mother eats other cereals	0.677	0.468	0.659	0.475	0.544
Mother eats cakes or buns	0.869	0.338	0.890	0.313	0.320
Mother eats crispbreads	0.318	0.466	0.322	0.468	0.913
Mother eats biscuits	0.913	0.282	0.914	0.281	0.969
Mother eats chocolate bars	0.839	0.367	0.820	0.385	0.406
Mother eats pulses	0.239	0.427	0.294	0.457	0.047
Mother eats nuts	0.317	0.465	0.310	0.463	0.809
Mother eats bean curd	0.024	0.154	0.047	0.212	0.025
Mother eats tahini	0.024	0.154	0.043	0.204	0.059
Mother eats soya or similar non-meat	0.080	0.271	0.078	0.269	0.945
Mother eats chocolate	0.763	0.426	0.780	0.415	0.515
Mother eats sweets	0.595	0.491	0.533	0.500	0.050
Mother drinks diet drinks	0.755	0.430	0.700	0.459	0.056
Partner eats sausages or burgers	0.738	0.440	0.709	0.455	0.341
Partner eats pies or pastries	0.711	0.454	0.644	0.480	0.034
Partner eats meat	0.949	0.220	0.891	0.312	<0.001
Partner eats poultry	0.925	0.263	0.886	0.318	0.036
Partner eats offal	0.226	0.418	0.177	0.383	0.092
Partner eats white fish	0.807	0.394	0.814	0.390	0.820
Partner eats oily fish	0.533	0.499	0.523	0.501	0.775
Partner eats shellfish	0.232	0.422	0.183	0.388	0.096
Partner eats fried food	0.759	0.428	0.714	0.453	0.131
Partner eats cabbage	0.888	0.316	0.900	0.301	0.579
Partner eats carrots	0.918	0.275	0.918	0.275	0.979
Partner eats other vegetables	0.969	0.174	0.973	0.163	0.736
Partner eats salad	0.868	0.339	0.923	0.268	0.018
Partner eats fresh fruit	0.881	0.324	0.872	0.335	0.687
Partner drinks tinned juice	0.227	0.419	0.191	0.394	0.212
Partner drinks pure non-tinned juice	0.714	0.452	0.682	0.467	0.298
Partner eats nuts	0.363	0.481	0.341	0.475	0.507
Partner drinks diet drink	0.566	0.496	0.556	0.498	0.777
Mother eats mostly white bread	0.575	0.494	0.549	0.499	0.411
Partner eats mostly white bread	0.780	0.415	0.750	0.434	0.384

© 2014 The Author(s).
The Economic Journal published by John Wiley & Sons Ltd on behalf of Royal Economic Society.

Table B1

(*Continued*)

	(1) Mother is homozygous for the common allele at rs1229984		(2) Mother carries at least one rare allele at rs1229984		(3) t-test
	Mean	SD	Mean	SD	p-value
Mother eats mostly brown/granary bread	0.446	0.497	0.475	0.500	0.367
Partner eats mostly brown/granary bread	0.641	0.480	0.653	0.478	0.770
Mothers eats mostly wholemeal bread	0.506	0.500	0.506	0.501	1.000
Partner eats mostly wholemeal bread	0.612	0.487	0.580	0.495	0.465
Mothers eats mostly chappatis	0.013	0.114	0.008	0.088	0.457
Partner eats mostly chappatis	0.055	0.228	0.020	0.140	0.124
Mother does not usually eat any bread	0.021	0.143	0.031	0.175	0.257
Partner does not usually eat any bread	0.075	0.264	0.041	0.199	0.264
Mother has takeaway meals	0.704	0.457	0.738	0.440	0.242
Partner has takeaway meals	0.689	0.463	0.687	0.465	0.954
Mother is vegetarian or vegan	0.131	0.338	0.157	0.365	0.233
Partner is vegetarian or vegan	0.033	0.178	0.064	0.246	0.013
Mother buys organic vegetables	0.306	0.461	0.296	0.457	0.737
Mother buys organic meat	0.143	0.350	0.148	0.356	0.831
Mother buys other organic foods	0.038	0.191	0.035	0.184	0.809
Parental attitudes					
Mother's attitude to breastfeeding [ranging from 4 to 23]‡	16.204	3.279	16.458	3.198	0.228
Partner's attitude to breastfeeding [ranging from 6 to 22]‡	15.591	2.679	15.731	2.951	0.461
Proportion agreeing (mother)					
Should pick up crying baby	0.483	0.500	0.532	0.500	0.129
Regular feed and sleep pattern is important	0.903	0.296	0.900	0.301	0.852
Should always be fed when they are hungry	0.942	0.233	0.933	0.251	0.536
Babies need stimulation to develop well	0.977	0.150	0.984	0.126	0.467
Babies should not be disturbed too much	0.550	0.498	0.560	0.497	0.735
Parents should adapt lives to baby's demands	0.523	0.500	0.552	0.498	0.365
Baby should fit into parents' routine	0.576	0.494	0.571	0.496	0.884
Babies should develop naturally	0.429	0.495	0.417	0.494	0.730
It is important to talk to a baby	0.999	0.025	0.996	0.063	0.069
Cuddling baby is very important	0.999	0.032	0.996	0.063	0.195
Proportion agreeing (partner):					
Should pick up crying baby	0.397	0.489	0.427	0.496	0.387
Regular feed and sleep pattern is important	0.918	0.274	0.949	0.221	0.113
Should always be fed when they are hungry	0.870	0.336	0.882	0.323	0.618
Babies need stimulation to develop well	0.978	0.146	0.962	0.191	0.125
Babies should not be disturbed too much	0.590	0.492	0.640	0.481	0.147
Parents should adapt lives to baby's demands	0.635	0.481	0.626	0.485	0.787
Baby should fit into parents' routine	0.502	0.500	0.498	0.501	0.900
Babies should develop naturally	0.411	0.492	0.500	0.501	0.010
It is important to talk to a baby	0.997	0.051	1.000	0.000	0.458
Cuddling baby is very important	0.997	0.053	1.000	0.000	0.436
Attitude to fatherhood score [ranging from 8 to 45]	38.477	5.183	38.596	5.788	0.758
Work and parenthood score [ranging from 1 to 12]	8.751	2.029	8.675	2.060	0.635

© 2014 The Author(s).
The Economic Journal published by John Wiley & Sons Ltd on behalf of Royal Economic Society.

Table B1

(*Continued*)

	(1) Mother is homozygous for the common allele at rs1229984		(2) Mother carries at least one rare allele at rs1229984		(3) t-test
	Mean	SD	Mean	SD	p-value
Religious beliefs					
Mother believes in a divine power	0.503	0.500	0.539	0.499	0.264
Partner believes in a divine power	0.360	0.480	0.408	0.493	0.149
Mother feels helped by divine power	0.339	0.474	0.377	0.486	0.209
Partner feels helped by divine power	0.240	0.427	0.271	0.445	0.308
Mother appeals to God for help if in trouble	0.474	0.499	0.506	0.501	0.323
Partner appeals to God for help if in trouble	0.352	0.478	0.426	0.496	0.028
Household characteristics					
Home is mortgaged/owned	0.796	0.403	0.829	0.377	0.191
Total number of rooms [ranging from 0 to 18]	4.942	1.507	4.879	1.417	0.508
Use of garden or yard	0.950	0.218	0.973	0.161	0.084
Working phone in home	0.920	0.271	0.933	0.251	0.461
Use of car by mum or partner	0.922	0.268	0.963	0.190	0.015
House has damp condensation or mould	0.481	0.500	0.448	0.498	0.295
Partner is father of child	0.994	0.074	0.996	0.061	0.699
Partner lives with mum	0.952	0.214	0.970	0.171	0.178
Age of partner [ranging from 16 to 60]	30.963	5.602	30.643	5.349	0.378
Marital status (eight weeks' gestation)	0.796	0.403	0.841	0.367	0.076
Total number of persons (eight weeks' gestation)	2.929	1.093	2.901	1.031	0.675
Pregnancy					
Seen doctor for possible infertility	0.131	0.337	0.136	0.343	0.815
Used treatments to help conceive	0.033	0.179	0.051	0.220	0.128
Has previously been pregnant	0.660	0.474	0.655	0.476	0.868
Has previously had a miscarriage	0.206	0.404	0.217	0.413	0.663
Has previously had an abortion or termination	0.134	0.340	0.098	0.299	0.099
Has previously had a stillbirth	0.008	0.088	0.011	0.106	0.530
Previous child born alive but died later	0.013	0.111	0.011	0.106	0.874
Mother's age when first pregnant [ranging from 12 to 42]	24.986	4.868	24.978	4.719	0.977
This pregnancy was intentional	0.730	0.444	0.780	0.415	0.074
Mother happy with pregnancy when first pregnant	0.717	0.450	0.756	0.431	0.177
Motherhood means personal sacrifice	0.705	0.456	0.667	0.472	0.184
Mother is currently happy with pregnancy	0.888	0.316	0.925	0.265	0.062
Partner is happy about pregnancy	0.869	0.338	0.867	0.340	0.928
Partner's first reaction was supportive	0.838	0.368	0.861	0.347	0.332
Partner is currently supportive	0.897	0.304	0.911	0.286	0.483
Mother already knew a lot about pregnancy	0.617	0.486	0.644	0.480	0.388
Self-induced vomiting for weight loss prior to pregnancy	0.053	0.224	0.073	0.261	0.156
Self-induced vomiting during this pregnancy	0.010	0.102	0.015	0.124	0.453
Laxative use for weight loss prior to pregnancy	0.042	0.200	0.035	0.183	0.593
Laxative use during this pregnancy	0.003	0.054	0.000	0.000	0.381
Mother had pain relief during labour	0.873	0.333	0.867	0.340	0.798
Mother had caesarean section	0.103	0.303	0.080	0.272	0.259
Partner was with mother during labour	0.882	0.322	0.884	0.320	0.912
Partner was with mother during delivery	0.854	0.353	0.873	0.334	0.429
Mother intends to work after child's birth	0.471	0.499	0.464	0.500	0.823

© 2014 The Author(s).
The Economic Journal published by John Wiley & Sons Ltd on behalf of Royal Economic Society.

Table B1
(*Continued*)

	(1) Mother is homozygous for the common allele at rs1229984		(2) Mother carries at least one rare allele at rs1229984		(3) t-test
	Mean	SD	Mean	SD	p-value
Mother's and partner's physical health					
Partner is well (eight weeks' gestation)	0.963	0.190	0.955	0.208	0.524
Mother is well prior to pregnancy	0.926	0.261	0.934	0.249	0.659
Mother is well in first trimester	0.407	0.491	0.457	0.499	0.114
Mother is well in second trimester	0.754	0.431	0.766	0.424	0.668
Mother is well in third trimester	0.766	0.423	0.776	0.417	0.697
Partner is well (eight month post-birth)	0.968	0.176	0.962	0.192	0.614
Height of mother (in cm)	164.183	6.767	164.057	6.960	0.772
Weight of mother pre-pregnancy (in kg)	62.086	11.030	60.483	9.620	0.025
Mother visited dentist in preg (measured post-preg)	0.771	0.420	0.842	0.366	0.055
Mother: nausea in first trimester	0.706	0.456	0.737	0.441	0.272
Mother: vomiting in first trimester	0.417	0.493	0.390	0.489	0.388
Mother: diarrhoea in first trimester	0.172	0.378	0.170	0.376	0.923
Mother: vaginal bleeding in first trimester	0.158	0.365	0.181	0.386	0.314
Mother: jaundice in first trimester	0.001	0.025	0.000	0.000	0.687
Mother: urinary infection in first trimester	0.048	0.213	0.062	0.241	0.299
Mother: influenza in first trimester	0.084	0.278	0.081	0.273	0.850
Mother: rubella in first trimester	0.000	0.014	0.000	0.000	0.816
Mother: thrush in first trimester	0.089	0.284	0.108	0.311	0.286
Mother: genital herpes in first trimester	0.001	0.038	0.000	0.000	0.537
Mother: other infection in first trimester	0.047	0.212	0.062	0.241	0.284
Mother: any infection in first trimester	0.232	0.422	0.266	0.443	0.202
Mother: injury or shock in first trimester	0.046	0.211	0.078	0.268	0.023
Mother: sugar in urine in first trimester	0.020	0.139	0.039	0.193	0.036
Mother: X-ray in first trimester	0.018	0.132	0.031	0.174	0.125
Mother: amniocentesis in first trimester	0.006	0.078	0.004	0.062	0.654
Mother: chorionic villus Sampling in first trimester	0.009	0.092	0.008	0.088	0.886
Mother: Spina Bifida test in first trimester	0.125	0.331	0.140	0.347	0.505
Mother: ultrasound scan in first trimester	0.265	0.441	0.310	0.463	0.111
Mother: admitted to hospital in first trimester	0.037	0.188	0.012	0.107	0.034
Mother: nausea in second trimester	0.364	0.481	0.391	0.489	0.393
Mother: vomiting in second trimester	0.220	0.414	0.234	0.424	0.581
Mother: diarrhoea in second trimester	0.310	0.462	0.246	0.432	0.032
Mother: vaginal bleeding in second trimester	0.043	0.202	0.039	0.194	0.773
Mother: jaundice in second trimester	0.001	0.029	0.000	0.000	0.643
Mother: urinary infection in second trimester	0.057	0.232	0.031	0.174	0.081
Mother: cold in second trimester	0.401	0.490	0.367	0.483	0.277
Mother: influenza in second trimester	0.056	0.229	0.039	0.194	0.257
Mother: rubella in second trimester	0.000	0.000	0.000	0.000	-
Mother: thrush in second trimester	0.132	0.338	0.102	0.303	0.162
Mother: genital herpes in second trimester	0.003	0.054	0.008	0.088	0.178
Mother: other infection in second trimester	0.053	0.224	0.055	0.228	0.900
Mother: any infection in second trimester	0.253	0.435	0.211	0.409	0.132
Mother: injury or shock in second trimester	0.076	0.265	0.070	0.256	0.731
Mother: sugar in urine in second trimester	0.128	0.335	0.117	0.322	0.600
Mother: X-ray in second trimester	0.009	0.093	0.027	0.163	0.003
Mother: amniocentesis in second trimester	0.018	0.132	0.016	0.124	0.812

© 2014 The Author(s).
The Economic Journal published by John Wiley & Sons Ltd on behalf of Royal Economic Society.

Table B1

(*Continued*)

	(1) Mother is homozygous for the common allele at rs1229984		(2) Mother carries at least one rare allele at rs1229984		(3) t-test
	Mean	SD	Mean	SD	p-value
Mother: chorionic villus Sampling in second trimester	0.008	0.087	0.004	0.063	0.506
Mother: spina bifida test in second trimester	0.232	0.422	0.293	0.456	0.026
Mother: ultrasound in second trimester	0.427	0.495	0.430	0.496	0.937
Mother: headache in second trimester	0.604	0.489	0.574	0.495	0.335
Mother: backache in second trimester	0.789	0.408	0.754	0.432	0.185
Mother: varicose veins in second trimester	0.144	0.351	0.148	0.356	0.842
Mother: admitted to hospital in second trimester	0.066	0.248	0.055	0.228	0.490
Mother's physical activity					
Mother exercises at least once a week	0.693	0.461	0.615	0.488	0.008
Mother usually walks	0.263	0.440	0.206	0.405	0.045
Mother usually cycles	0.009	0.096	0.012	0.108	0.693
Mother usually uses public transport	0.074	0.262	0.054	0.227	0.244
Mother usually uses the car	0.721	0.449	0.774	0.419	0.063
Mother: jogging	0.019	0.138	0.012	0.108	0.395
Mother: aerobics	0.050	0.217	0.051	0.221	0.905
Mother: ante-natal exercise	0.242	0.428	0.251	0.434	0.732
Mother: keep fit exercises	0.136	0.343	0.142	0.349	0.790
Mother: yoga	0.030	0.170	0.032	0.175	0.868
Mother: squash	0.005	0.069	0.004	0.063	0.859
Mother: tennis or badminton	0.030	0.170	0.032	0.176	0.852
Mother: swimming	0.453	0.498	0.420	0.495	0.310
Mother: brisk walking	0.753	0.431	0.719	0.450	0.215
Mother: weight training	0.006	0.079	0.008	0.089	0.746
Mother: cycling	0.061	0.239	0.071	0.258	0.501
Mother: other exercise	0.083	0.276	0.105	0.307	0.219
Parental mental health					
Bachman self-esteem score [−4 to 2]	0.015	0.987	0.035	0.969	0.760
Mother's self-perceived change score [11–35]§	23.860	2.734	24.206	2.811	0.050
Mother's self-perceived feel good score [7–30]§	16.799	3.866	16.659	3.877	0.576
Mother's perception of partner's change score [9–35]§	20.938	1.674	20.928	1.853	0.930
Mother's perception of partner's feel good score [7–31]§	13.734	4.026	13.498	3.863	0.373
Partner's self-perceived change score [9–35]§	20.647	2.309	20.832	2.069	0.251
Partner's self-perceived feel good score [7–30]§	15.791	3.806	15.690	3.786	0.705
Partner's perception of mother's change score [10–35]§	20.368	1.538	20.257	1.538	0.309
Partner's perception of mother's feel good score [7–32]§	14.901	3.839	14.557	4.082	0.208
Mother: interpersonal awareness score [7–28]	18.324	4.659	18.835	4.866	0.085
Mother: Need for approval score [8–32]	25.836	3.581	26.073	3.122	0.296
Mother: separation anxiety score [8–32]	16.211	4.612	16.275	4.939	0.827
Mother: timidity score [8–32]	20.644	4.503	21.111	4.362	0.101
Mother: fragile inner-self score [5–20]	8.701	2.919	8.748	2.954	0.799
Mother: total interpersonal sensitivity score [36–140]	89.704	15.828	91.034	15.818	0.185

© 2014 The Author(s).
The Economic Journal published by John Wiley & Sons Ltd on behalf of Royal Economic Society.

Table B1
(*Continued*)

	(1) Mother is homozygous for the common allele at rs1229984		(2) Mother carries at least one rare allele at rs1229984		(3) t-test
	Mean	SD	Mean	SD	p-value
Partner: interpersonal awareness score [7–28]	16.229	4.854	16.467	4.683	0.487
Partner: need for approval score [0–32]	24.540	4.319	24.507	4.344	0.914
Partner: separation anxiety score [0–32]	14.692	4.386	14.519	4.567	0.577
Partner: timidity score [3–32]	18.896	4.697	18.877	4.794	0.955
Partner: fragile inner-self score [5–20]	8.187	2.725	8.267	2.635	0.680
Partner: Total interpersonal sensitivity score [29–138]	82.456	16.270	82.519	15.482	0.956
Mother: pre-17 life event score [0–63]	8.751	8.324	8.685	8.096	0.901
Partner: pre-17 life event score [0–74]	9.890	8.700	10.552	9.027	0.272
Partner's affection score (mother reported) [6–30]¶	11.340	4.087	11.363	3.913	0.930
Partner's affection score (partner reported) [6–30]¶	10.929	3.905	10.970	4.033	0.886
Partner's aggression score (mother reported) [3–15]¶	10.082	1.750	9.945	1.671	0.224
Partner's aggression score (partner reported) [3–15]¶	9.915	1.862	9.782	1.927	0.328
Maternal use of medication					
Any medications used since start of pregnancy	0.705	0.456	0.702	0.458	0.918
Medication for nausea in first trimester	0.043	0.202	0.064	0.245	0.093
Medication for heartburn in first trimester	0.071	0.256	0.042	0.200	0.070
Medication for vomiting in first trimester	0.031	0.173	0.049	0.216	0.101
Medication for anxiety in first trimester	0.005	0.068	0.004	0.061	0.843
Medication for infection in first trimester	0.082	0.274	0.095	0.293	0.461
Medication for migraine in first trimester	0.124	0.329	0.106	0.309	0.396
Medication for sleeping in first trimester	0.008	0.087	0.000	0.000	0.154
Medication for pain in first trimester	0.123	0.328	0.102	0.304	0.321
Medication for allergies in first trimester	0.030	0.170	0.030	0.171	0.979
Medication for skin condition in first trimester	0.073	0.260	0.075	0.265	0.886
Medication for bleeding in first trimester	0.006	0.075	0.000	0.000	0.221
Medication for depression in first trimester	0.005	0.069	0.000	0.000	0.258
Medication for piles in first trimester	0.022	0.146	0.023	0.149	0.934
Medication for constipation in first trimester	0.054	0.225	0.038	0.191	0.260
Medication for cough in first trimester	0.052	0.222	0.064	0.245	0.388
Medication for other reasons in first trimester	0.067	0.250	0.057	0.232	0.533
Taking iron in first trimester	0.197	0.397	0.177	0.382	0.427
Taking zinc in first trimester	0.015	0.120	0.015	0.122	0.956
Taking calcium in first trimester	0.032	0.175	0.045	0.208	0.222
Taking folic acid in first trimester	0.086	0.280	0.094	0.292	0.645
Taking vitamins in first trimester	0.160	0.367	0.198	0.399	0.107
Taking other supplements or diet foods in first trimester	0.030	0.170	0.019	0.138	0.327
Ever used homeopathic medicine (trimester 1)	0.103	0.304	0.102	0.303	0.933
Taking aspirin in first trimester	0.042	0.201	0.053	0.224	0.396
Taking paracetamol in first trimester	0.550	0.498	0.491	0.501	0.060
Taking codeine or anadin in first trimester	0.024	0.154	0.015	0.122	0.342
Taking sleeping tablets in first trimester	0.004	0.063	0.000	0.000	0.302
Taking tranquiliser in first trimester	0.003	0.051	0.008	0.087	0.141

© 2014 The Author(s).
The Economic Journal published by John Wiley & Sons Ltd on behalf of Royal Economic Society.

Table B1
(*Continued*)

	(1) Mother is homozygous for the common allele at rs1229984		(2) Mother carries at least one rare allele at rs1229984		(3) t-test
	Mean	SD	Mean	SD	p-value
Number of medications used in first trimester [0–17]	1.350	1.446	1.330	1.370	0.825
Medication for nausea in second trimester	0.025	0.156	0.008	0.088	0.082
Medication for heartburn in second trimester	0.367	0.482	0.332	0.472	0.251
Medication for vomiting in second trimester	0.016	0.126	0.004	0.063	0.125
Medication for anxiety in second trimester	0.007	0.083	0.012	0.108	0.382
Medication for infection in second trimester	0.107	0.309	0.090	0.287	0.389
Medication for migraine in second trimester	0.077	0.267	0.070	0.256	0.692
Medication for sleeping disorder in second trimester	0.032	0.177	0.031	0.174	0.925
Medication for pain in second trimester	0.155	0.362	0.117	0.322	0.105
Medication for allergies in second trimester	0.046	0.211	0.035	0.185	0.400
Medication for skin condition in second trimester	0.108	0.311	0.145	0.352	0.070
Medication for bleeding in second trimester	0.003	0.058	0.000	0.000	0.352
Medication for depression in second trimester	0.007	0.081	0.004	0.063	0.606
Medication for piles in second trimester	0.077	0.266	0.090	0.287	0.450
Medication for constipation in second trimester	0.070	0.255	0.063	0.243	0.650
Medication for cough in second trimester	0.076	0.266	0.094	0.292	0.313
Medication for other reason in second trimester	0.107	0.309	0.094	0.292	0.514
Taken iron in last second trimester	0.426	0.494	0.422	0.495	0.907
Taken zinc in last second trimester	0.013	0.115	0.012	0.108	0.812
Taken calcium in last second trimester	0.032	0.175	0.035	0.185	0.751
Taken folic acid in second trimester	0.193	0.395	0.191	0.394	0.947
Taken vitamins in second trimester	0.114	0.318	0.117	0.322	0.873
Taken other supplements in second trimester	0.026	0.159	0.027	0.163	0.904
Ever use homeopathic medicine (trimester 2)	0.156	0.363	0.162	0.369	0.788
Taken aspirin use in second trimester	0.028	0.166	0.027	0.164	0.942
Taken paracetamol in second trimester	0.434	0.496	0.361	0.481	0.022
Taken codein or anadin in second trimester	0.017	0.130	0.024	0.152	0.459
Taken sleeping pill in second trimester	0.007	0.086	0.012	0.108	0.431
Taken tranquiliser in second trimester	0.002	0.041	0.004	0.063	0.412
Number of medications used in second trimester [0–11]	1.661	1.568	1.673	1.542	0.906
Taken sleeping pill since birth (measured at eight months)	0.015	0.120	0.004	0.064	0.176
Taken cannabis since birth (measured at eight months)	0.030	0.169	0.025	0.155	0.652
Taken tranquilliser since birth (measured at eight months)	0.007	0.082	0.008	0.090	0.804
Taken anti-depressant since birth (measured at eight months)	0.043	0.204	0.029	0.167	0.271
Taken hormone tablet since birth (measured at eight months)	0.009	0.092	0.016	0.127	0.210
Taken antibiotic since birth (measured at eight months)	0.267	0.442	0.246	0.432	0.469
Taken painkiller since birth (measured at eight months)	0.818	0.386	0.803	0.398	0.555
Taken amphetamine since birth (measured at eight months)	0.005	0.071	0.004	0.064	0.833

© 2014 The Author(s).
The Economic Journal published by John Wiley & Sons Ltd on behalf of Royal Economic Society.

Table B1
(*Continued*)

	(1) Mother is homozygous for the common allele at rs1229984		(2) Mother carries at least one rare allele at rs1229984		(3) t-test
	Mean	SD	Mean	SD	p-value
Taken the pill since birth (measured at eight months)	0.515	0.500	0.516	0.501	0.958
Taken opiate or cocaine since birth (meas. at eight months)	0.002	0.045	0.000	0.000	0.486
Taken anticonvulsant since birth (measured at eight months)	0.003	0.051	0.004	0.064	0.672
Taken steroid since birth (measured at eight months)	0.017	0.128	0.008	0.090	0.303
Taken iron since birth (measured at eight months)	0.208	0.406	0.225	0.419	0.526
Taken vitamin since birth (measured at eight months)	0.254	0.435	0.254	0.436	0.999
Taken other substance since birth (measured at eight months)	0.184	0.387	0.168	0.375	0.532
Parental substance use					
Mother smoked pre-pregnancy	0.299	0.458	0.270	0.445	0.310
Mother ever smoked	0.567	0.495	0.523	0.500	0.164
Father ever smoked	0.763	0.425	0.744	0.437	0.483
Partner smokes (at 18 weeks' gestation)	0.341	0.474	0.346	0.477	0.855
Partner's number of cigarettes (at eight months) [0–60]	3.712	7.698	3.846	7.598	0.794
Mother smoked cannabis during pregnancy	0.023	0.149	0.019	0.138	0.726
Mother smoked cannabis in six months prior to pregnancy	0.043	0.204	0.039	0.194	0.735
Mother used amphetamine during pregnancy	0.001	0.028	0.004	0.062	0.125
Mother used barbiturate during pregnancy	0.000	0.020	0.000	0.000	0.745
Mother used crack during pregnancy	0.000	0.000	0.000	0.000	-
Mother used cocaine during pregnancy	0.001	0.025	0.000	0.000	0.690
Mother used heroin during pregnancy	0.000	0.014	0.000	0.000	0.819
Mother used methadone during pregnancy	0.000	0.014	0.000	0.000	0.818
Mother used ecstasy during pregnancy	0.003	0.051	0.000	0.000	0.700
Mother used other drug during pregnancy	0.002	0.040	0.004	0.062	0.402
Mother used hard drugs during pregnancy	0.003	0.058	0.008	0.087	0.270
Partner smoked cannabis in six months prior to pregnancy	0.117	0.322	0.104	0.307	0.654
Partner smoked cannabis in first trimester	0.095	0.294	0.090	0.288	0.843
Partner used amphetamine in first trimester	0.006	0.080	0.000	0.000	0.235
Partner used barbiturate in first trimester	0.001	0.036	0.000	0.000	0.596
Partner used crack in first trimester	0.001	0.028	0.000	0.000	0.682
Partner used cocaine in first trimester	0.002	0.046	0.000	0.000	0.502
Partner used heroin in first trimester	0.001	0.032	0.000	0.000	0.636
Partner used methadone in first trimester	0.001	0.032	0.000	0.000	0.636
Partner used ecstasy in first trimester	0.005	0.073	0.000	0.000	0.613
Partner used other in first trimester	0.010	0.098	0.015	0.120	0.490
Partner used hard drugs in first trimester	0.015	0.123	0.014	0.117	0.871
Mother used ganja in last two months of pregnancy	0.018	0.132	0.013	0.112	0.560
Mother used ganja since birth (measured at eight weeks)	0.026	0.158	0.013	0.112	0.210

© 2014 The Author(s).
The Economic Journal published by John Wiley & Sons Ltd on behalf of Royal Economic Society.

Table B1

(*Continued*)

	(1) Mother is homozygous for the common allele at rs1229984		(2) Mother carries at least one rare allele at rs1229984		(3) t-test
	Mean	SD	Mean	SD	p-value
Mother used hard drugs in last two months of pregnancy	0.001	0.033	0.000	0.000	0.606
Mother used hard drugs since birth (measured at eight weeks)	0.003	0.057	0.008	0.090	0.208
Mother's use of chemicals during pregnancy					
Disinfectant	0.874	0.332	0.841	0.366	0.117
Bleach	0.845	0.362	0.841	0.366	0.872
Window cleaner	0.621	0.485	0.601	0.491	0.521
Carpet cleaner	0.376	0.484	0.358	0.480	0.548
Oven or drain cleaner	0.416	0.493	0.380	0.486	0.244
Dry cleaning fluid	0.059	0.235	0.063	0.243	0.790
Turps or white spirit	0.217	0.412	0.188	0.392	0.261
Paint stripper	0.058	0.234	0.048	0.214	0.481
House paint or varnish	0.326	0.469	0.277	0.448	0.094
Weed killer	0.076	0.264	0.063	0.243	0.433
Pesticide	0.276	0.447	0.255	0.436	0.450
Aerosol or spray	0.823	0.382	0.815	0.389	0.754
Hair dye or bleach	0.163	0.370	0.144	0.352	0.396
Hair removal cream	0.110	0.312	0.100	0.300	0.607
Air freshener	0.697	0.460	0.657	0.476	0.165
Use of other chemicals	0.061	0.240	0.066	0.249	0.736
Electrical mixer	0.539	0.499	0.551	0.498	0.695
Hoover use	0.966	0.181	0.958	0.200	0.507
Floor polisher	0.056	0.230	0.023	0.149	0.020
Electrical iron	0.964	0.186	0.951	0.216	0.270
Electrical hair appliance	0.876	0.330	0.879	0.326	0.867
Electrical typewriter	0.161	0.368	0.204	0.404	0.069
Photocopier or fax	0.407	0.491	0.453	0.499	0.142
PC or VDU	0.409	0.492	0.479	0.501	0.024
Power tool	0.059	0.235	0.045	0.208	0.361
Sunbed or lamp	0.015	0.121	0.019	0.136	0.601
Microwave	0.777	0.417	0.767	0.423	0.719
Other electrical equipment	0.099	0.299	0.083	0.276	0.391
Dental amalgam	0.013	0.112	0.015	0.121	0.780
Ceramic or enamel	0.019	0.135	0.015	0.121	0.648
Dry cleaning	0.021	0.143	0.033	0.179	0.178
Electroplating	0.001	0.028	0.000	0.000	0.642
Glue	0.258	0.438	0.283	0.451	0.359
Leather working	0.004	0.061	0.000	0.000	0.310
Fabric and textile	0.167	0.373	0.169	0.376	0.936
Dye	0.043	0.203	0.026	0.159	0.163
Insecticide	0.112	0.316	0.110	0.314	0.918
Plastics	0.039	0.194	0.022	0.147	0.155
Metal cleaner	0.219	0.414	0.191	0.394	0.275
Petrol	0.390	0.488	0.375	0.485	0.629
Paint	0.287	0.453	0.246	0.432	0.145
Photo chemical	0.015	0.121	0.018	0.135	0.650
ELEC wiring	0.031	0.173	0.029	0.169	0.893

© 2014 The Author(s).
The Economic Journal published by John Wiley & Sons Ltd on behalf of Royal Economic Society.

Table B1
(*Continued*)

	(1) Mother is homozygous for the common allele at rs1229984		(2) Mother carries at least one rare allele at rs1229984		(3) t-test
	Mean	SD	Mean	SD	p-value
Machining	0.046	0.210	0.048	0.214	0.903
Soldering	0.006	0.077	0.004	0.061	0.629
Radiation	0.025	0.158	0.029	0.169	0.690
Social support					
Mother's social network score (at 12 weeks' gestation) [5–29]††	23.615	3.690	23.605	4.047	0.965
Partner's social network score (at 18 weeks' gestation) [1–29]††	22.503	3.890	22.605	3.880	0.706
Mother's social support score (at 12 weeks' gestation) [0–30]††	19.963	4.914	20.358	5.115	0.223
Partner's social support score (at 18 weeks' gestation) [1–30]††	17.930	4.859	18.205	4.889	0.415
Neighbourhood characteristics					
Mother thinks neighbourhood is a good place to live	0.933	0.251	0.928	0.259	0.770
Partner thinks neighbourhood is a good place to live	0.932	0.251	0.918	0.275	0.440
Mother: people in neighbourhood visit	0.550	0.498	0.587	0.493	0.234
Mother: people in neighbourhood argue with mother	0.044	0.204	0.026	0.159	0.164
Mother: people in neighbourhood look after children	0.202	0.401	0.238	0.427	0.151
Mother: people in neighbourhood keep to themselves	0.822	0.382	0.807	0.396	0.520
Mother visits others in neighbourhood	0.496	0.500	0.524	0.500	0.368
Mother argues with people in neighbourhood	0.037	0.189	0.022	0.148	0.208
Mother looks after neighbours' children	0.216	0.411	0.257	0.438	0.113
Mother keeps to herself	0.807	0.394	0.792	0.407	0.526
Partner: people in neighbourhood visit	0.516	0.500	0.549	0.499	0.370
Partner: people in neighbourhood argue with mother	0.051	0.221	0.031	0.174	0.206
Partner: people in neighbourhood look after children	0.211	0.408	0.313	0.465	0.001
Partner: people in neighbourhood keep to themselves	0.881	0.324	0.870	0.337	0.673
Partner visits others in neighbourhood	0.378	0.485	0.421	0.495	0.229
Partner argues with people in neighbourhood	0.041	0.199	0.036	0.187	0.725
Partner looks after neighbours' children	0.155	0.362	0.179	0.384	0.387
Partner keeps to himself	0.871	0.335	0.845	0.363	0.293
Mother is worried about possible burglary	0.845	0.361	0.863	0.345	0.454
Mother is worried about possible mugging/robbery	0.655	0.475	0.664	0.473	0.758
Mother is worried about possible sex assault	0.640	0.480	0.656	0.476	0.597
Mother is worried about possible vandalism to home	0.700	0.458	0.714	0.453	0.627
Partner is worried about possible burglary	0.871	0.335	0.896	0.306	0.322
Partner is worried about possible mugging/robbery	0.496	0.500	0.537	0.500	0.274

© 2014 The Author(s).
The Economic Journal published by John Wiley & Sons Ltd on behalf of Royal Economic Society.

Table B1

(*Continued*)

	(1) Mother is homozygous for the common allele at rs1229984		(2) Mother carries at least one rare allele at rs1229984		(3) t-test
	Mean	SD	Mean	SD	p-value
Partner is worried about possible sex assault	0.226	0.418	0.212	0.410	0.655
Partner is worried about possible vandalism to home	0.737	0.441	0.773	0.420	0.258
Mother thinks neighbourhood is lively	0.569	0.495	0.553	0.498	0.604
Mother thinks neighbourhood is friendly	0.932	0.252	0.940	0.238	0.624
Mother thinks neighbourhood is noisy	0.511	0.500	0.515	0.501	0.903
Mother thinks neighbourhood is clean	0.915	0.278	0.914	0.282	0.918
Mother thinks neighbourhood is attractive	0.833	0.373	0.827	0.379	0.797
Mother thinks neighbourhood is polluted or dirty	0.285	0.451	0.274	0.447	0.712
Partner thinks neighbourhood is lively	0.319	0.467	0.231	0.439	0.507
Partner thinks neighbourhood is friendly	0.881	0.324	0.846	0.376	0.704
Partner thinks neighbourhood is noisy	0.244	0.430	0.231	0.439	0.913
Partner thinks neighbourhood is clean	0.825	0.381	0.769	0.439	0.612
Partner thinks neighbourhood is attractive	0.710	0.455	0.538	0.519	0.190
Partner thinks neighbourhood is polluted or dirty	0.132	0.339	0.231	0.439	0.314

Notes. All variables are measured during pregnancy, unless otherwise stated. All variables are binary unless otherwise stated, indicating the range of the values, for example, [0–30]. *The educational indicators are: less than ordinary (O) level (ref), O-level only, advanced (A) level that permits higher educational study and university degree. The social class variables use the standard (reversed, so that higher values correspond to higher social classes) UK classification of social class based on occupation (professional, managerial/technical, non-manual skilled, manual skilled, semi-skilled and unskilled). Family income is an average of two observations (when the child is aged 3 and 4) and is in 1995 prices. It is adjusted for family size and composition (equalised) using the OECD equivalence scale to allow for a comparison of incomes for all households. EPDS and CCEI refer to the mother's Edinburgh Postnatal Depression Score and the Crown-Crisp Experimental Index. EPDS indicates to what extent the mother is at risk of perinatal depression; CCEI captures a broader definition of mental health, measuring general anxiety, depression and somaticism. Higher scores mean the mother is more affected. †Mother's diet is measured at 32 weeks' gestation; partner's diet is measured at 18 weeks' gestation. ‡'Attitude to breastfeeding', 'attitude to fatherhood' and 'work and parenthood' are derived from multiple questions, with higher scores indicating more positive attitudes. §The self-perceived change and feel good scores relate to physical, emotional and behavioural changes during early pregnancy, with higher scores indicating more positive changes. ||The partner's affection (aggression) scores are derived from multiple questions, where higher scores indicate less affection (aggression). ††The social network and social support scores are derived from multiple questions, where higher scores indicate a larger network and more support.

© 2014 The Author(s).

Appendix C. Parental Responsive Investments

Table C1

Potential Parental Investments in Response to Child Development

	(1) Coefficient on average number of units during pregnancy	(2) SE	(3) Mean	(4) First-stage F-statistic	(5) N
Child diet and nutrition					
Baby has fruit juice (four weeks)	0.004	(0.042)	0.125	14.651	2,620
Baby has vitamins (four weeks)	0.004	0.034	0.071	14.651	2,620
Baby has glucose solution (four weeks)	0.010	0.011	0.006	14.651	2,620
Baby has cereal (four weeks)	0.008	0.023	0.019	14.651	2,620
Baby has other diet supplements (four weeks)	−0.013	0.041	0.100	14.651	2,620
Child had formula (six months)	0.149**	0.070	0.825	13.790	2,546
Child had follow-on milk (six months)	−0.075	0.049	0.093	13.790	2,546
Child had soya milk (six months)	0.007	0.024	0.029	13.790	2,546
Child had goat's milk (six months)	0.001	0.001	0.001	13.790	2,546
Child had hypo-allergenic formula (six months)	−0.011	0.013	0.002	13.790	2,546
Child had cow's milk (six months)	−0.079	0.060	0.198	13.790	2,546
Child had plain baby rice (six months)	−0.033	0.039	0.887	13.790	2,546
Child had flavoured baby rice (six months)	0.032	0.064	0.382	13.790	2,546
Child had other cereal (six months)	0.024	0.051	0.818	13.790	2,546
Child had sweetened rusks (six months)	−0.023	0.059	0.225	13.790	2,546
Child had plain rusks (six months)	−0.017	0.065	0.612	13.790	2,546
Child had bread or toast (at six months)	−0.022	0.065	0.375	13.790	2,546
Child had biscuits (six months)	−0.011	0.057	0.223	13.790	2,546
Child had prepared savoury meat (six months)	0.066	0.054	0.832	13.790	2,546
Child had prepared savoury fish (six months)	−0.102	0.072	0.359	13.790	2,546
Child had prepared savoury veg (six months)	−0.033	0.039	0.898	13.790	2,546
Child had prepared fruit pudding (six months)	0.011	0.043	0.873	13.790	2,546
Child had prepared milk pudding (six months)	−0.036	0.065	0.583	13.790	2,546
Child had home-cooked egg (six months)	−0.022	0.047	0.145	13.790	2,546
Child had home-cooked meat (six months)	0.031	0.067	0.479	13.790	2,546
Child had home-cooked fish (six months)	−0.090	0.068	0.303	13.790	2,546
Child had home-cooked potatoes (six months)	−0.037	0.039	0.883	13.790	2,546
Child had home-cooked veg (six months)	−0.058	0.047	0.816	13.790	2,546
Child had home-made fruit puddings (six months)	−0.035	0.068	0.463	13.803	2,539
Child had home-made milk puddings (six months)	−0.055	0.059	0.189	13.790	2,546
Child had Coca Cola or Pepsi (six months)	−0.001	0.012	0.011	13.790	2,546
Child had other fizzy drink (six months)	0.014*	0.007	0.008	13.790	2,546
Child had apple juice (six months)	0.016	0.059	0.246	13.790	2,546
Child had a little alcohol (six months)	0.005	0.015	0.024	13.790	2,546
Child had blackcurrant/rose hip syrup (six months)	−0.068	0.068	0.344	13.790	2,546
Child had other fruit drink (six months)	0.028	0.067	0.557	13.790	2,546
Child had herbal drink (six months)	−0.138*	0.072	0.495	13.790	2,546

© 2014 The Author(s).
The Economic Journal published by John Wiley & Sons Ltd on behalf of Royal Economic Society.

Table C1

(*Continued*)

	(1) Coefficient on average number of units during pregnancy	(2) SE	(3) Mean	(4) First-stage F-statistic	(5) *N*
Child had gripe water (six months)	0.033	0.067	0.542	13.790	2,546
Child had tea (six months)	0.042	0.029	0.072	13.790	2,546
Child had coffee (six months)	0.021**	0.010	0.010	13.790	2,546
Child had raw fruit (six months)	−0.047	0.067	0.418	13.790	2,546
Child had crisps (six months)	−0.027	0.028	0.022	13.790	2,546
Child had chocolates (six months)	0.011	0.054	0.207	13.790	2,546
Child had sweets (six months)	0.005	0.007	0.012	13.790	2,546
Child had raw veg (six months)	−0.092	0.061	0.167	13.790	2,546
Child had packet soup (15 months)	−0.060	0.038	0.082	22.616	2,482
Child had canned soup (15 months)	0.019	0.057	0.429	23.377	2,489
Child had liver (15 months)	0.018	0.048	0.198	22.857	2,490
Child had kidney (15 months)	0.014	0.026	0.067	23.050	2,489
Child had shell fish (15 months)	0.025	0.030	0.089	22.752	2,491
Child had baked beans (15 months)	0.011	0.030	0.927	22.662	2,495
Child had green peas (15 months)	0.021	0.029	0.938	22.637	2,494
Child had other legumes (15 months)	−0.102*	0.061	0.270	22.069	2,476
Child had yoghurt (15 months)	−0.005	0.017	0.965	21.959	2,488
Child had fig (15 months)	−0.008	0.025	0.040	21.574	2,474
Child had raw apple (15 months)	−0.020	0.039	0.833	22.754	2,485
Child had other raw fruit (15 months)	−0.019	0.018	0.965	22.002	2,488
Child had raw carrot (15 months)	−0.029	0.058	0.454	22.798	2,483
Child had other raw vegetables (15 months)	0.003	0.039	0.171	23.304	2,316
Child had nuts (15 months)	−0.000	0.036	0.082	22.161	2,483
Child had crisps (15 months)	−0.050	0.054	0.637	22.108	2,485
Child had other savoury snacks (15 months)	0.003	0.055	0.672	22.242	2,476
Child had chocolate (15 months)	0.081	0.052	0.841	22.011	2,486
Child had mints (15 months)	0.004	0.027	0.041	22.383	2,478
Child had sweets (15 months)	−0.053	0.057	0.302	22.748	2,480
Child ever had gravy or soy sauce (15 months)	0.059	0.051	0.805	22.553	2,484
Child ever had salt (15 months)	0.035	0.043	0.229	22.663	2,485
Child ever had herbs (15 months)	−0.029	0.058	0.430	22.604	2,467
Child ever had spices (15 months)	0.002	0.043	0.188	22.605	2,482
Child ever had tomato ketchup (15 months)	0.020	0.050	0.275	22.244	2,484
Child ever had other sauce (15 months)	−0.007	0.037	0.117	22.723	2,338
Child ever had sugar (15 months)	−0.012	0.053	0.275	21.705	2,467
Child ever had smoked or cured food (15 months)	0.057	0.058	0.410	22.573	2,490
Child ever had microwave meal (15 months)	0.050**	0.023	0.064	22.609	2,489
Child ever had BBQ food (15 months)	0.052	0.039	0.144	22.381	2,488
Child ever had sports drink (15 months)	0.011***	0.003	0.012	22.376	2,492
Number of meals a day (six months) [ranging from 1 to 4]	−0.008	0.057	2.903	13.775	2,539
Number of meals a day (38 months) [ranging from 1 to 4]	−0.025	0.029	2.944	12.080	2,324
Feeding difficulties (six months)	0.072	0.066	0.353	13.226	2,528
Cereal added to child's bottle (six months)	−0.018	0.020	0.022	14.223	2,506
Sugar added to child's food/bottle (six months)	0.012	0.034	0.071	13.558	2,532
Child uses dummy (six months)	0.105	0.071	0.463	13.790	2,546
Immunisation and other treatment					
Began to immunise baby at four weeks	0.019	0.025	0.044	14.241	2,608
BCG (tuberculosis) immunisation (six months)	−0.018	0.018	0.008	13.790	2,546

© 2014 The Author(s).
The Economic Journal published by John Wiley & Sons Ltd on behalf of Royal Economic Society.

Table C1

(*Continued*)

	(1) Coefficient on average number of units during pregnancy	(2) SE	(3) Mean	(4) First-stage F-statistic	(5) *N*
DTP immun. incl. whooping cough (six months)	−0.026	0.038	0.916	13.790	2,546
DT immun. excl. whooping cough (six months)	−0.020	0.029	0.044	13.790	2,546
Polio immunisation (six months)	0.061	0.055	0.829	13.790	2,546
Hib (meningitis) immunisation (six months)	−0.006	0.056	0.239	13.790	2,546
Fluoride treatment (six months)	−0.017	0.021	0.013	10.560	2,307
Child has vitamins (24 months)	0.054	0.056	0.246	11.543	2,383
Child has vitamins (38 months)	−0.027	0.061	0.291	12.476	2,364
(Night-time) interactions†					
Partner ever feeds baby at night (four weeks)	−0.009	0.063	0.668	13.894	2,561
Feed baby when wakes at night (four weeks)	0.014	0.019	0.988	14.704	2,581
Give baby water when wakes at night (four weeks)	0.107**	0.047	0.139	14.704	2,581
Cuddle baby when wakes at night (four weeks)	−0.019	0.036	0.886	14.704	2,581
Give baby dummy when wakes at night (four weeks)	0.070	0.066	0.405	14.704	2,581
Baby to mother's bed when wakes (four weeks)	0.034	0.062	0.668	14.704	2,581
Nappy change when wakes at night (four weeks)	0.009	0.025	0.966	14.704	2,581
Other activity when baby wakes (four weeks)	−0.001	0.024	0.040	14.704	2,581
Ever wake baby for feed (four weeks)	0.005	0.054	0.764	14.793	2,599
Give baby milk when wakes at night (six months)	−0.075	0.068	0.557	13.331	2,362
Give baby other drink when wakes (six months)	0.158**	0.070	0.307	13.331	2,362
Cuddle baby when wakes at night (six months)	0.078	0.063	0.748	13.331	2,362
Give baby dummy when wakes at night (six months)	0.107	0.069	0.429	13.331	2,362
Baby to mum's bed when wakes (six months)	0.014	0.066	0.487	13.331	2,362
Nappy change when wakes at night (six months)	0.136*	0.072	0.644	13.331	2,362
Other activity when baby wakes (six months)	0.029	0.036	0.111	13.331	2,362
Partner interaction score (42 months) [0–36]	−0.464	0.816	21.778	10.989	2,223
Mother interaction score (42 months) [0–36]	−0.172	0.589	28.966	12.812	2,328
Other person interaction score (42 months) [0–36]	−0.754	0.749	18.219	25.219	1,102
Doctor and dentist visits					
Child uses toothbrush (15 months)	0.010	0.028	0.953	22.580	2,483
Child uses toothbrush (24 months)	0.005	0.008	0.997	11.160	2,376
Child uses toothbrush (38 months)	−0.034**	0.014	0.978	12.476	2,364
Child uses toothpaste (15 months)	−0.025	0.038	0.896	22.429	2,482
Child uses toothpaste (24 months)	0.027	0.023	0.989	10.782	2,373
Child uses toothpaste (38 months)	−0.026**	0.011	0.979	12.476	2,364
Child ever visited dentist (38 months)	−0.118**	0.048	0.829	12.476	2,364
Mother took baby to health clinic (four weeks)	−0.051	0.059	0.689	14.337	2,593
Doctor called to home for child (six months)	−0.000	0.060	0.271	13.353	2,534
Doctor called to home for child (18 months)	0.128*	0.075	0.378	11.722	2,474
Doctor called to home for child (30 months)	0.126*	0.067	0.280	14.059	2,373
Specialist checked child (24 months)	0.020	0.052	0.188	12.694	2,358
Child had surgery visit (30 months)	−0.015	0.055	0.814	13.079	2,364
Child had routine check with doctor (30 months)	−0.037	0.055	0.172	12.726	2,332
Parenting and teaching scores‡					
Child's activity score (six months) [0–20]	−0.067	0.330	14.480	13.804	2,543
Child's activity score (30 months) [0–29]	0.032	0.424	18.589	14.381	2,392
Child's activity score (42 months) [0–28]	0.172	0.364	18.724	12.818	2,324
Mother's parenting score (six months) [0–12]	−0.135	0.193	10.542	13.668	2,539
Mother's parenting score (18 months) [6–51]	−0.254	0.572	40.860	13.025	2,481
Mother's parenting score (24 months) [20–40]	0.462	0.394	34.547	10.632	2,324

© 2014 The Author(s).
The Economic Journal published by John Wiley & Sons Ltd on behalf of Royal Economic Society.

Table C1

(*Continued*)

	(1) Coefficient on average number of units during pregnancy	(2) SE	(3) Mean	(4) First-stage F-statistic	(5) *N*
Mother's parenting score (38 months) [4–30]	−0.291	0.405	25.250	12.614	2,356
Partner's parenting score (six months) [10–30]	0.311	0.496	23.599	14.612	2,493
Partner's parenting score (18 months) [0–40]	1.057	0.944	24.486	11.804	2,406
Partner's parenting score (38 months) [0–30]	−0.097	0.700	21.447	11.186	2,248
Mother's teaching score (30 months) [0–8]	−0.007	0.159	6.637	14.167	2,380
Mother's teaching score (42 months) [0–8]	0.190	0.179	6.993	12.817	2,325
Child's toy score (24 months) [5–36]	−0.532	0.497	23.512	9.978	2,317
Child's toy score (42 months) [1–9]	−0.097	0.090	8.180	12.817	2,325
Maternal care score (18 months) [0–24]	0.196	0.683	20.044	15.367	2,655
Maternal overprotective score (18 months) [0–20]	−0.342	0.553	6.288	15.367	2,655
Maternal enjoyment score (eight months) [0–15]	−0.254	0.230	13.243	13.240	2,506
Maternal confidence score (eight months) [4–18]	−0.033	0.249	15.088	13.240	2,506
Maternal bonding score (eight months) [4–33]	−0.286	0.401	28.331	13.240	2,506
Time spent doing different activities					
TV is on for most of the day (30 months)	0.033	0.066	0.463	14.043	2,224
TV is on for most of the day (42 months)	0.052	0.083	0.475	9.271	2,166
Hours per week spent in car (38 months) [0–14]	−0.084	0.211	3.928	12.854	2,331
Hours per week spent outdoors (38 months) [0–14]	0.811	0.500	9.652	12.394	2,319
Hours per week spent watching TV (38 months) [0–14]	−0.181	0.488	7.487	12.872	2,327
Hours per week spent week/other kids (38 months) [0–14]	0.009	0.464	11.668	12.411	2,312
Mother has nights out each week (eight months)	−0.010	0.052	0.196	12.954	2,417
Mother worried that child may					
Get accident (18 months)	0.093	0.065	0.717	13.032	2,477
Get meningitis (18 months)	0.057	0.065	0.639	13.019	2,472
Get asthma (18 months)	0.073	0.065	0.350	11.574	2,429
Get fits (18 months)	0.005	0.049	0.162	12.996	2,470
Be mentally handicapped (18 months)	0.011	0.030	0.052	13.020	2,471
Get AIDS (18 months)	−0.005	0.035	0.098	12.818	2,470
Worried about any aspect of behaviour (42 months)	−0.123*	0.063	0.194	13.224	2,259
Household characteristics[§]					
Other children in house (six months)	−0.003	0.067	0.540	13.178	2,529
Other children in house (18 months)	−0.077	0.070	0.574	12.343	2,471
Older children in house (18 months)	−0.038	0.068	0.538	12.285	2,468
Younger children in house (18)	−0.042	0.035	0.047	12.441	2,456
Total number of household members (eight months) [1–14]	−0.117	0.143	3.793	13.131	2,461
Financial difficulties score (eight months) [0–15]	−0.147	0.467	2.752	13.348	2,497
Child care					
Expect to use partner (at 32 weeks' gestation)	0.075*	0.041	0.163	15.699	2,670
Expect to use family (at 32 weeks' gestation)	0.051	0.049	0.188	15.469	2,661
Expect to use child minder (at 32 weeks' gestation)	−0.004	0.039	0.116	15.314	2,648
Expect to use nanny (at 32 weeks' gestation)	−0.017	0.024	0.038	14.801	2,647
Expect to use nursery (at 32 weeks' gestation)	0.028	0.028	0.046	15.386	2,620
Expect to use other (at 32 weeks' gestation)	−0.015	0.016	0.017	15.465	2,663
Partner regularly looks after child (15 months)	0.032	0.050	0.778	21.799	2,473
Grandparent regularly looks after child (15 months)	0.103*	0.057	0.443	21.799	2,473

© 2014 The Author(s).
The Economic Journal published by John Wiley & Sons Ltd on behalf of Royal Economic Society.

Table C1

(*Continued*)

	(1) Coefficient on average number of units during pregnancy	(2) SE	(3) Mean	(4) First-stage F-statistic	(5) *N*
Other relative regularly looks after child (15 months)	−0.014	0.034	0.099	21.799	2,473
Friend regularly looks after child (15 months)	−0.056	0.043	0.097	21.799	2,473
Childminder regularly looks after child (15 months)	0.010	0.039	0.142	21.799	2,473
Nanny regularly looks after child (15 months)	−0.022	0.030	0.062	21.799	2,473
Nursery regularly looks after child (15 months)	0.056**	0.024	0.061	21.799	2,473
Someone else regularly looks after child (15 months)	0.020	0.016	0.017	21.799	2,473
Partner regularly looks after child (24 months)	0.062	0.064	0.720	11.060	2,363
Grandparent regularly looks after child (24 months)	0.171**	0.081	0.447	11.060	2,363
Other relative regularly looks after child (24 months)	0.021	0.042	0.116	11.060	2,363
Friend regularly looks after child (24 months)	−0.001	0.047	0.140	11.060	2,363
Childminder regularly looks after child (24 months)	−0.011	0.051	0.147	11.060	2,363
Nanny regularly looks after child (24 months)	0.019	0.035	0.066	11.060	2,363
Nursery regularly looks after child (24 months)	0.041	0.043	0.101	11.060	2,363
Other person regularly looks after child (24 months)	0.017	0.019	0.012	11.060	2,363
Partner regularly looks after child (38 months)	0.009	0.057	0.766	12.476	2,364
Grandparents regularly look after child (38 months)	0.136*	0.071	0.429	12.476	2,364
Other relative regualrly looks after child (38 months)	−0.038	0.044	0.094	12.476	2,364
Friend regularly looks after child (38 months)	−0.016	0.043	0.122	12.476	2,364
Childminder regularly looks after child (38 months)	−0.083	0.051	0.094	12.476	2,364
Nanny regularly looks after child (38 months)	0.041	0.033	0.073	12.476	2,364
Nursery regularly looks after child (38 months)	0.052	0.067	0.359	12.476	2,364
Other person regularly looks after child (38 months)	−0.111	0.072	0.319	12.476	2,364
Number of types of child care (38 months) [0–6]	−0.010	0.157	2.255	12.476	2,364
Social support‖					
Mother's social support score (eight weeks) [0–30]	0.334	0.646	20.374	15.599	2,578
Partner's social support score (eight weeks) [0–30]	−0.647	0.607	19.422	20.743	1,991
Mother's social help score (eight weeks) [0–24]	−0.696	0.522	16.306	15.596	2,577
Partner's social help at home (eight weeks) [0–27]	−1.384**	0.621	15.298	21.418	1,998
Partner's social help with child (eight weeks) [0–35]	0.871	0.802	20.669	21.418	1,998

Notes. All variables are measured after the child is born, unless otherwise stated. All variables are binary unless otherwise stated, indicating the range of the variable, for example, [0–30]. The coefficients (column 1) and standard errors (column 2) denote the estimates from an IV regression of the effect of alcohol exposure *in utero* on the outcome of interested listed in the first column, where the mother's *ADH1B* is used as the instrument. Column 3 shows the mean of the outcome of interest, column 4 shows the first-stage F-statistic, and column 5 shows the sample size for each analysis. All 'score-variables' are derived from multiple questions. *p < 0.10, **p < 0.05, ***p < 0.01. †The interaction scores indicate the frequency and type of interactions with the child, with higher scores indicating more interactions. ‡The activity scores measure activities such as going to the park, supermarket, visiting friends etc., with higher scores indicating more activity. The parenting scores measure activities such as reading stories, eating together, cuddling, slapping, singing to the child etc., where higher scores indicate better parenting. The teaching scores capture activities such as teaching numbers, rhymes, shapes, politeness etc., where higher scores indicate more teaching. The toy scores capture the number and types of toys the child has, such as push/pull, co-ordination toys, lego, books etc., where higher scores indicate more toys. The maternal care and overprotective scores measure the relationship between the mother and her mother (e.g. whether the mother's mother was friendly, cold, controlling, affectionate etc.). The maternal enjoyment, confidence and bonding scores capture whether the mother enjoys looking after, is confident and bonds with her baby. §The financial difficulties score measures how difficult it is to afford food, clothing, heating, rent etc. ‖The social support score measures the extent of support available to the mother and partner (including emotional support, sharing happiness, relying on each other etc.). The social help scores measure the extent to which the mother and partner receive help with the baby, doing shopping, cleaning, cooking, washing etc.

© 2014 The Author(s).
The Economic Journal published by John Wiley & Sons Ltd on behalf of Royal Economic Society.

Appendix D. Robustness Checks

Table D1

Robustness Checks Using Key Stage 1 as the Outcome Variable and the Number of Alcoholic Units Consumed as the Treatment Variable

	(1) Coefficient on average number of units during pregnancy	(2) SE	(3) First-stage F-statistic	(4) *N*
Panel (a): controlling for additional alcohol-related covariates				
1. Replicates the results from Table 5	−0.245**	0.114	16.366	2,433
2. Includes maternal smoking during pregnancy as covariate	−0.242**	0.114	15.909	2,431
3. Excludes child *ADH1B* (i.e. only the principal components)	−0.182**	0.084	33.526	2,433
4. Includes binary indicators for maternal post-natal alcohol intake when the child was 8, 21, 33 and 47 months old	−0.204	0.138	7.922	1,861
5. Includes binary indicators for the child's own alcohol intake at 157, 166 and 185 months	−0.140	0.119	11.816	1,125
6. Includes mother's partner's and parents' alcohol consumption	−0.232**	0.113	14.216	2,035
Panel (b): controlling for covariates specified in Appendix B				
7. Includes all 'standard' covariates	−0.217*	0.122	10.856	1,551
8. Same as model (7), but using (single) multivariate imputation for missing values on the covariate to obtain the same sample size as the original specification	−0.273**	0.128	11.030	2,433
9. Includes mother's tea, coffee and milk intake, eight weeks' gestation	−0.299**	0.150	11.373	2,345
10. Includes mother's diet and nutrition, 32 weeks' gestation	−0.198*	0.105	14.975	2,241
11. Includes mother's attitude to parenting	−0.209*	0.121	12.881	2,254
12. Includes mother's religious believes	−0.234**	0.118	14.168	2,385
13. Includes household characteristics	−0.319**	0.130	12.888	2,292
14. Includes variables related to the mother's pregnancy	−0.221	0.142	7.479	1,914
15. Includes mother's physical health during pregnancy	−0.294*	0.153	9.274	1,273
16. Includes mothers' physical activity during pregnancy	−0.211**	0.095	28.892	2,280
17. Includes mother's mental health during pregnancy	−0.160	0.116	12.127	2,200
18. Includes mother's use of medication during pregnancy	−0.206**	0.100	15.147	2,246
19. Includes mother's substance use during pregnancy	−0.199*	0.113	12.940	2,096
20. Includes mother's use of chemicals	−0.183*	0.101	16.751	2,426
21. Includes mother's social support network	−0.225*	0.117	14.856	2,296
22. Includes mother's perception of neighbourhood	−0.222**	0.108	16.577	2,339

Notes. All estimates come from separate IV regressions. All regressions also control for the 10 ancestry-informative principal components and the child's *ADH1B*, apart from specification 3 that only controls for the principal components. Robust standard errors are presented in column (2). *$p < 0.10$, **$p < 0.05$, ***$p < 0.01$.

© 2014 The Author(s).
The Economic Journal published by John Wiley & Sons Ltd on behalf of Royal Economic Society.
