# Supplementary material for: Alcohol Exposure In Utero and Child Academic Achievement*
Source: Econ J (London). 2014 May 23;124(576):634–67. doi: 10.1111/ecoj.12144 (PMC4243528; doi:10.1111/ecoj.12144)
Supplement: Supplementary file 2 [file ecoj0124-0634-sd2.pdf]

Alcohol Exposure *In Utero* and Child Academic Achievement

Stephanie von Hinke Kessler Scholder, George L. Wehby, Sarah Lewis, Luisa Zuccolo

The Avon Longitudinal Study of Parents and Children

Pregnant women living in one of three Bristol-based health districts in the former County of Avon with an expected delivery date between April 1991 and December 1992 were eligible to be enrolled in the Avon Longitudinal Study of Parents and Children (ALSPAC). Around 14,000 pregnant women were initially recruited. Detailed information has been collected using self-administered questionnaires, data extraction from medical notes, linkage to routine information systems and at research clinics. Ethical approval for the study was obtained from the ALSPAC Ethics and Law Committee (IRB00003312) and Local Research Ethics Committees.

ALSPAC has collected vast amounts of information on the cohort members and their families, linked to administrative records on health, education, economic, criminal, and neighbourhood data, as well as an extensive biobank.

Please see http://www.bristol.ac.uk/alspac/researchers/data-access/ for full information on how to obtain data from ALSPAC. Any proposal to obtain, use, and collect data has to be directed to the ALSPAC executive committee using a Research Proposal Form. The executive committee will generally reply within two weeks.

All questionnaires and survey information can be searched via indexed files, available on the ALSPAC website: http://www.bristol.ac.uk/alspac/researchers/data-access/datadictionary/.

After approval of the proposal, any standard existing data will be provided by your assigned "data buddy". Research using SNP genotype data requires a legally-binding agreement between the University of Bristol and the host institution. Once the proposal is approved, ALSPAC will provide the specific documents to be completed, signed and returned.

For more information about ALSPAC, see:

- www.bristol.ac.uk/alspac
- Fraser, A., Macdonald-Wallis, C., Tilling, K., Boyd, A., Golding, J., Davey Smith, G., Henderson, J., Macleod, J., Molloy, L., Ness, A., Ring, S., Nelson, S. and Lawlor, D.A. (2013). 'Cohort profile: the Avon Longitudinal Study of Parents and Children: ALSPAC mothers cohort', *International Journal of Epidemiology*, Vol. 42(1), pp. 97-110.
- Boyd, A., Golding, J., Macleod, J., Lawlor, D.A., Fraser, A., Henderson, J., Molloy, L., Ness, A., Ring, S. and Davey Smith, G. (2013). 'Cohort Profile: The 'Children of the 90s' – the index offspring of the Avon Longitudinal Study of Parents and Children', *International Journal of Epidemiology*, Vol. 42(1), pp. 111–127.
